# The human ribosomal DNA array is composed of highly homogenized tandem clusters

**DOI:** 10.1101/gr.275838.121

**Published:** 2021-11

**Authors:** Yutaro Hori, Akira Shimamoto, Takehiko Kobayashi

**Affiliations:** 1 Institute for Quantitative Biosciences, the University of Tokyo, Tokyo 133-0032, Japan;; 2 Faculty of Pharmaceutical Sciences, Sanyo-Onoda City University, Sanyo Onoda, Yamaguchi 756-0884, Japan

## Abstract

The structure of the human ribosomal DNA (rDNA) cluster has traditionally been hard to analyze owing to its highly repetitive nature. However, the recent development of long-read sequencing technology, such as Oxford Nanopore sequencing, has enabled us to study the large-scale structure of the genome. Using this technology, we found that human cells have a quite regular rDNA structure. Although each human rDNA copy has some variations in its noncoding region, contiguous copies of rDNA are similar, suggesting that homogenization through gene conversion frequently occurs between copies. Analysis of rDNA methylation by Nanopore sequencing further showed that all the noncoding regions are heavily methylated, whereas about half of the coding regions are clearly unmethylated. The ratio of unmethylated copies, which are speculated to be transcriptionally active, was lower in individuals with a higher rDNA copy number, suggesting that there is a mechanism that keeps the active copy number stable. In addition, the rDNA in progeroid syndrome patient cells with reduced DNA repair activity had more unstable copies compared with control normal cells, although the rate was much lower than previously reported using a fiber-FISH method. Collectively, our results clarify the view of rDNA stability and transcription regulation in human cells, indicating the presence of mechanisms for both homogenizations to ensure sequence quality and maintenance of active copies for cellular functions.

The ribosomal DNA (rDNA) cluster is the most abundant repetitive gene region in eukaryotic cells. In the budding yeast *Saccharomyces cerevisiae*, the structure and function of rDNA have been well studied, establishing rDNA as a unique region in the genome. Each unit (9.2 kb) of rDNA includes two coding regions, 35S precursor ribosomal RNA (rRNA) and 5S rRNA genes, and two noncoding intergenic spacer (IGS) regions between the genes. The units tandemly repeat (approximately 150 times) in Chromosome XII (Petes 1979; Kobayashi et al. 1998). A unique feature of the yeast rDNA is that it has a system to maintain the quality and quantity of the sequence to fulfill the huge demand for ribosomes in the cell (Gangloff et al. 1996; for review, see Kobayashi 2011). The rDNA tends to lose copies through recombination between them because of their repetitive nature and highly activated transcription. To maintain quantity, therefore, the rDNA amplifies more copies when the number is reduced (Kobayashi et al. 1998). As a result, the rDNA is continually undergoing contraction and expansion and thus is one of the most unstable regions in the genome (Kobayashi 2008).

The DNA-binding protein Fob1 is a key player in the amplification reaction (Kobayashi 2003). It induces recombination for amplification by inhibiting replication at the replication fork barrier (RFB) (Supplemental Fig. S1). The inhibition induces DNA double-strand breaks at a relatively high frequency, and the repair process increases the number of copies by unequal sister chromatid recombination (Weitao et al. 2003; Burkhalter and Sogo 2004; Kobayashi et al. 2004). Recombination is also regulated by noncoding transcription (E-pro transcription) through cohesion dissociation (Kobayashi and Ganley 2005). In terms of quality control of rDNA, the sequences are always homogenized; that is, a copy with mutations is excluded by a Fob1-dependent recombination mechanism, such as gene conversion and contraction of the copies (Ganley and Kobayashi 2007). In fact, the rDNA sequences in the budding yeast are known to be relatively uniform although about half the copies are not transcribed (Ganley and Kobayashi 2011). Therefore, we speculate that active recombination in the rDNA maintains the integrity that ensures intact rRNA and the ribosome.

There is another face of such unstable rDNA—namely, it induces cellular senescence in budding yeast (Ganley and Kobayashi 2014). For example, in the *fob1* mutant, the rDNA is stable with less recombination and the mutant's life span is extended by ∼60% (Defossez et al. 1999; Takeuchi et al. 2003). In contrast, in the *sir2* mutant, in which E-pro transcription is enhanced and the rDNA copy number frequently changes, life span is shortened by ∼50% (Kaeberlein et al. 1999; Saka et al. 2013). Because the rDNA is a large unstable region in the genome, its instability may affect the stability of the whole-genome and thereby influence life span (i.e., the rDNA theory of aging) (Kobayashi 2008).

Although we have good knowledge about yeast rDNA and its extra coding functions for aging, there is limited information on human rDNA. One reason is that the human rDNA unit (∼43 kb) is much larger than the yeast rDNA unit (∼9.2 kb), and it includes many small repetitive sequences in the noncoding region. Although the Human Genome Project declared its completion in 2003, it was difficult to assemble the rDNA into its actual composition using the relatively short “reads” that were obtained from the sequencing technology of those days. However, the recent development of DNA polymerase-independent long-read sequencing technologies, such as the Oxford Nanopore Technologies or Pacific Biosciences (PacBio) systems, has made it possible to assemble complete sequences of the unexplored regions (Miga et al. 2020).

The human rDNA comprises 100–500 copies in a cell (Agrawal and Ganley 2018; Parks et al. 2018). Each unit of rDNA consists of the 45S precursor RNA gene (45S rDNA), whose transcript is processed into mature 18S, 5.8S, and 28S RNAs, and the IGS, which is filled with small repetitive sequences such as microsatellites and transposons (Fig. 1A). In the IGS, there are two typical repeats: the R repeat and Butterfly/Long repeat. The R repeat (∼680 bp, typically three copies) is located in the termination region of the 45S rRNA gene. It contains the Sal box that is associated with the transcription factor TTFI (Grummt et al. 1986). TTFI, similar to yeast Fob1, functions to inhibit the replication fork to avoid the collision of RNA and DNA polymerase (Akamatsu and Kobayashi 2015). The Butterfly/Long repeat (∼4500 bp, typically two copies), which is composed of a Long repeat, CT microsatellite, and Butterfly repeat, is located at approximately the center of the IGS (Fig. 1A; Gonzalez and Sylvester 2001; Agrawal and Ganley 2018).

**Figure 1.**
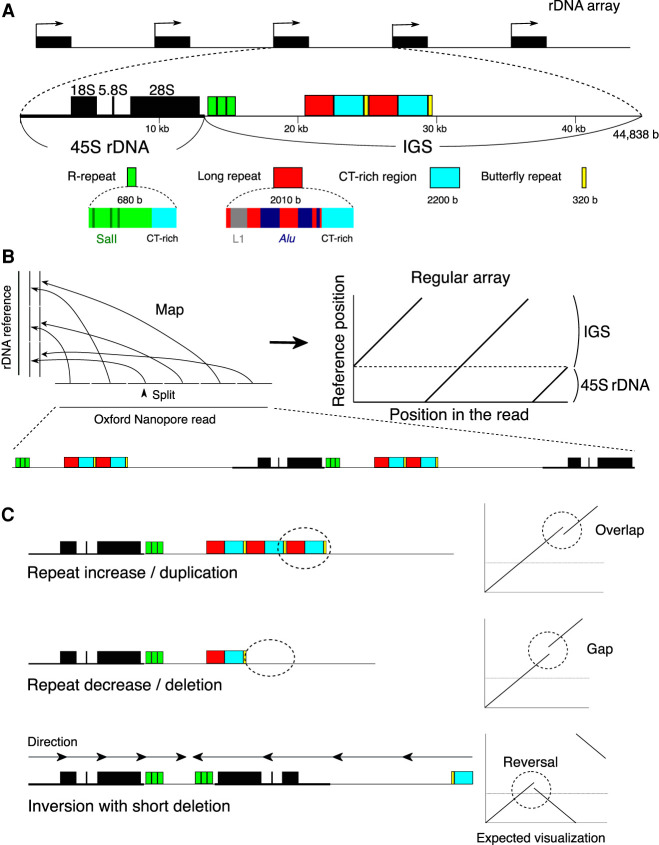
rDNA structure and strategy for visualizing rDNA. (*A*) Structure of rDNA. rDNA is largely divided into the coding 45S rRNA gene (45S rDNA) and the noncoding intergenic spacer (IGS). The IGS has repetitive sequences, such as microsatellite and transposable elements. Here, typical R and Long/Butterfly repeats are shown. (*B*) rDNA visualization strategy for Nanopore reads containing rDNA. First, the read is split into 300-nt sections, and each split read is mapped to the rDNA reference sequence. The structure is then reconstructed based on the position in the read and the mapped position in the reference. (*C*) Typical mutations and how they look in the visualization strategy. In this example, the reference sequence has three R and two Long/Butterfly repeats.

A previous study of rDNA composition in human cells by an in situ hybridization method (fiber-FISH) reported that many irregular units, such as palindromic inverted and incomplete units, account for ∼35% of the total copies in rDNA (Caburet et al. 2005). This high rate indicates that there is no effective recombination system to maintain rDNA homogeneity in human cells. In addition, the ratio of these noncanonical rDNA units was found to be increased in cells from progeroid syndrome patients, suggesting that human rDNA is also related to senescence. Because most progeroid syndromes, such as Werner syndrome and Bloom syndrome, are caused by mutations of the DNA repair machinery, it is plausible that the symptoms of these syndromes are caused by instability in the rDNA, which is thought to be one of the most unstable DNA regions in human, as in budding yeast (Carrero et al. 2016). Indeed, previous studies have suggested that rDNA copy number varies greatly in cells from Bloom syndrome patients, and palindromic structures have been observed in Werner syndrome cells (Schawalder et al. 2003; Killen et al. 2009). However, it is still unclear whether rDNA instability is an important factor in senescence in human. In terms of the relationship between rDNA and senescence in mammals, rDNA is also known to become methylated during the passage of life (Wang and Lemos 2019). The ratio of rDNA methylation works as a “clock” that tells the individual's age.

To reveal the detailed structure and integrity in the human rDNA cluster at the DNA sequence level, we developed a method to analyze rDNA-derived long reads obtained by an Oxford Nanopore sequencer.

## Results

### Long sequence reads reveal variations among rDNA copies

To determine the human rDNA structure, we analyzed both publicly available Oxford Nanopore whole-genome sequencing (WGS) data from the Human Pangenomics Project (HPGP, https://github.com/human-pangenomics/), and our in-house Cas9-enriched rDNA reads from Epstein-Barr virus (EBV) transformed B cells (Shafin et al. 2020) and primary fibroblast cells (Supplemental Table S1). Taking advantage of the high copy number of rDNA per cell, we modified the Cas9-enrichment strategy and established a protocol to construct a library at lower cost (Gilpatrick et al. 2020). In short, to enrich the rDNA fragments, we designed four guide RNAs around the 9500- to 9900-nt region from the start site of the coding region (45S rDNA) of the reference sequence; all four sequences were strictly conserved in human and mouse. We designed gRNAs in the coding region because this region is thought to have fewer mutations, and the relationship between two neighboring 45S rDNAs can be analyzed in a single read. Total DNA was dephosphorylated with CIP to avoid ligation to the sequencing adapter. The DNA was then digested by Cas9 ribonucleoprotein (RNP). Because only the Cas9-digested DNA fragments have a phosphorylated-5′ end, the sequencing adapters are specifically ligated to the fragments. In analyzing the HPGP genomic data, we removed reads shorter than 40,000 nt to eliminate those that were thought to come from rDNA-derived pseudogenes in non-rDNA genomic regions. For our in-house Cas9-enriched data, we analyzed the reads from DNA fragments in which both ends were digested with Cas9 RNP.

To determine the structure of rDNA, we developed a method that visualizes multiple copies of rDNA and the structural variation. In this method, the reads are split into 300 nt and mapped to an rDNA reference sequence (NCBI GenBank [https://www.ncbi.nlm.nih.gov/genbank/] accession number KY962518.1) using BWA-MEM aligner software suited for long-read mapping (Li 2013; Kim et al. 2018). Such a split can eliminate sequencing errors and nucleotide level variations that are not necessary for our structural analysis. The shorter split length increases not only resolution but also the effect of sequencing errors of the Nanopore sequencer and reduces mapping frequency. By testing several lengths of split reads, we found that a 300-nt split is long enough to accomplish high mapping frequency (Supplemental Fig. S2A). Each 300-nt split read (short line) is plotted based on its location in the original read and its mapped position in the reference sequence (Methods; Fig. 1A, left). Therefore, when the reads are the same as the reference, a continuous straight line is generated (Fig. 1B, right). If there is a deletion, duplication, or translocation, however, the line will be discontinuous (Fig. 1C). In selecting the reference sequence, we compared three different reference sequences of human rDNA (GenBank accession KY962518, U13369, and AL592188) by counting the number of gaps between successive split-and-mapped reads. If the distance between two neighboring mapped reads differed from the expected distance (300 nt) by more than 100 nt, we considered that the pair was gapped and we counted the number of gaps. We found that KY962518 had the least number of gaps for all samples and thus should be the most typical rDNA sequence as the reference (Supplemental Fig. S2B; Agrawal and Ganley 2018; Kim et al. 2018).

**Figure 2.**
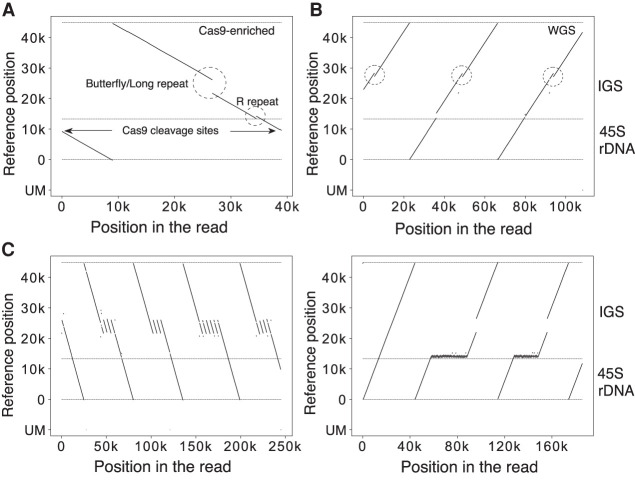
Visualization examples. (*A*,*B*) Representative visualization pattern of a read obtained from Cas9-enrichment (*A*) and whole-genome (*B*) sequencing. The vertical axis is the mapped position in the reference; the *horizontal* axis is the position in the Oxford Nanopore read. The horizontal dashed lines indicate the end of the reference and the border between 45S rDNA and IGS. The unmapped split reads are shown at the *bottom* (UM). The Sal box and the Butterfly/Long repeat region, which show variability among copies, are indicated (*A*). The same type of IGS variation is seen in all three rDNA copies (dashed circles) (*B*). (*C*) rDNA copies with an extremely long Butterfly/Long and R repeat. This kind of R repeat expansion was seen in four individuals (HG01258, HG02080, HG02257, PSCA0047); for all of them, multiple copies of R repeat expansion were detected. These sequences cannot be analyzed by short-read sequencers.

Figure 2A shows actual representative data of a Cas9-enriched read. The read (∼40 kb) had one copy of rDNA with a gap in the Butterfly/Long repeat and a duplication in the R repeat region. Figure 2B shows actual data from HPGP WGS sequencing. The length of this read was ∼110 kb, corresponding to two and a half tandem copies of rDNA, that is, IGS–45S–IGS–45S–IGS. From the visualization, we could identify that all the copies had the same duplicated regions in the Butterfly/Long repeat region. Therefore, this split-and-map visualizing method is robust and should work in the structural analysis of rDNA. A strong point of the long-read sequencer is that repeat structures of DNA can be analyzed; indeed, we were able to identify copies with extremely high repeat number variation in their IGS (Fig. 2C). In contrast, a weak point is that the fidelity of sequencing is not as high as that of short-read sequencers. But, as mentioned above, selecting an appropriate split length (300 nt) reduces this weakness and makes our structural analysis possible.

### R and Butterfly/Long repeats are highly variable between copies

**Figure 3.**
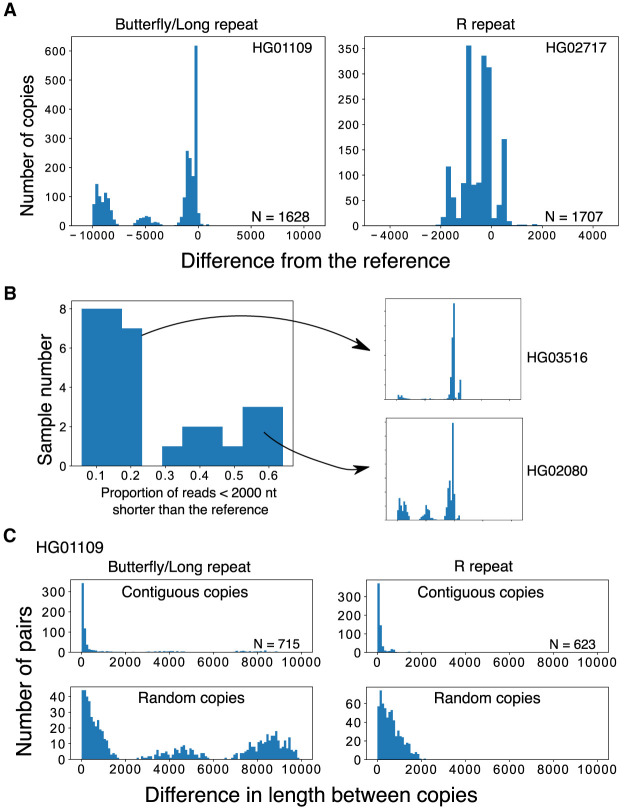
Variations in the IGS region. (*A*) Length distributions of the variable Butterfly/Long and R repeats of the rDNA IGS in WGS samples. Distributions are plotted based on the difference from the reference (0 indicates the reference length). In these samples, repeat number variations can clearly be seen in the discrete peaks. (*B*) Plot showing the proportion of reads with a Butterfly/Long repeat length shorter (<2000 nt) than the reference for each sample. The samples can clearly be divided into two categories with each category having a similar pattern of size distribution among individuals (Supplemental Fig. S2). Typical distributions of long (HG03516) and short (HG02080) types are plotted on the *right*. (*C*) Differences in the length of contiguous reads calculated for the R and Butterfly/Long repeats (*upper* panels). As a control, we calculated the differences in the length of the repeats of randomly picked copy pairs (*lower* panels).

By applying the split-and-map visualizing method to Nanopore data, we analyzed 39 samples (individuals) and identified variations in the Butterfly/Long and R repeat regions (Fig. 3A). By measuring the Butterfly/Long repeat length of each read in each individual, we could classify the distribution into two types based on the proportion of copies that were more than 2000 bases shorter than the reference (Fig. 3B; Supplemental Fig. 3A). These two types were also clearly differentiated by principal component analysis (PCA), confirming that our classification was not arbitrary (Supplemental Fig. S3B). In the shorter type, there were three discrete peaks and two of them were shorter than the reference (e.g., HG02080). In the longer type (e.g., HG03516), the smaller peaks were not obvious in many cases, and >70% of the copies were almost the same as the reference. All the Japanese samples belonged to the shorter type (*N* = 6: A0031, BSL2KA, PSCA0023, PSCA0047, PSCA0060, PSCA0517). Thus, there are differences among populations. In terms of the R repeat, the copy number varied from 0 to 4 (average 2–3 for most samples). As shown in Supplemental Figure S4, the variation among samples was much larger for the R repeat than for the Butterfly/Long repeat and there were no clear differences among populations.

### Contiguous copies have similar variation patterns

By comparing copies within reads that contain more than one copy of rDNA, the similarity between contiguous copies can be calculated. Specifically, we analyzed the differences in lengths of the R and Butterfly/Long repeat regions (Fig. 3C, upper panel). As a control, we also simulated the case in which two random copies are compared (Fig. 3C, lower panel; Supplemental Fig. S5). In all samples, the distributions of length difference between contiguous copies were clearly shorter than the randomized simulated control in both regions. These observations indicate that contiguous copies are more similar than noncontiguous ones. Therefore, this suggests that gene conversion and/or other recombination events, at least, occurs locally and homogenizes the sequences of these repeats.

### The rDNA is quite regular in human cells

Next, we analyzed larger-scale structural features of the rDNA. In a previous study, it was reported that human rDNA contains many noncanonical irregular copies, such as palindrome structures (Caburet et al. 2005). Such irregular structures were suggested by a fiber-FISH (DNA combing) assay, in which long rDNA spread on a slide glass is hybridized with two fluorescent probes for different sites in the rDNA, and fluorescence signals are detected by microscopy. As a result, noncanonical copies are identified by the irregularity of the aligned dotted signals. The study indicated that irregular rDNA copies account for ∼35% of total rDNA copies in a cell. Furthermore, this ratio was increased in cells from Werner syndrome patients (∼50%).

**Figure 4.**
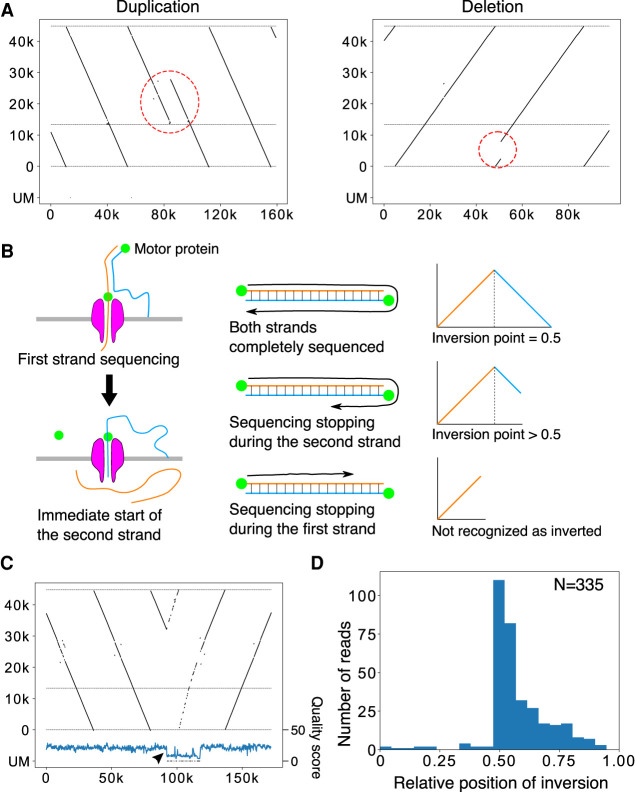
Large-scale structural variation in the rDNA array. (*A*) Representative reads with large-scale variation in the rDNA array. (*Left* panel) The R to Butterfly/Long repeat region is doubled. (*Right* panel) A large portion of rDNA in the 45S rDNA is deleted. (*B*) The mechanism of Nanopore template switching that causes artifactual “fake” palindromic reads. (*Left* panel) After the completion of first strand sequencing, second (complementary) strand sequencing sometimes occurs. (*Right* panel) When sequencing terminates randomly after strand switching, the resulting distribution of the inversion points in the reads should be seen in only the latter half of the reads. (*C*) A representative palindromic read. The structure and the end points of the read are similar before and after the inversion point, although the read is relatively long. The Phred quality scores plotted *below* were smoothed by binning and averaging. A sudden drop in quality score is observed just after the inversion point (arrowhead). (*D*) Plot of the relative position of inversion in each read for HPGP samples. The distribution is peaked at the center and heavily skewed to the latter half of reads, suggesting that most of the palindromic reads are artifacts.

Using long sequence reads, we expected to obtain a more accurate description of the larger-scale structure of rDNA. First, we screened the reads in which >10% of the split reads were mapped in the opposite direction to the majority of the split reads and labeled them as inverted reads. Then, using the split-and-map visualizing data, we measured the distances between the splits and compared them to the distance that was calculated from the reference sequence: if the repeat has an irregularly aligned or unusual structure, the distance between adjacent split reads will be larger and we can detect the difference. We did not use information stored within the CIGAR string, which reports a detailed alignment result, and the analysis was based solely on split-read alignments. We found that reads with such large gaps are rare; that is, most of the rDNA copies were beautifully tandemly aligned on the chromosomes (Supplemental Table S1). In fact, the rate of noncanonical copies excluding palindromic reads in the healthy samples was <1% (0% – 0.7%). Furthermore, in ∼30% (12/39) of the individual samples, no noncanonical copies were detected. The most common structural mutation was duplication from the R repeat to the Long repeat region (Fig. 3A, left; Supplemental Table S1). We consider this type of mutation to be a common variation because it is limited to the IGS region, where it is likely to have little impact on rRNA transcription. Another typical structural mutation was deletion (Fig. 4A, right).

We found many palindromic reads, but they are thought to be artifacts for the following reason. The Oxford Nanopore sequencer reads single-stranded DNA by separating double-stranded DNA at the pore (Fig. 4B). If the separation does not occur properly at the end of the first strand, sequencing of the second complementary strand may follow immediately (de Lannoy et al. 2017). Therefore, the resulting sequence read will look like a palindrome. Such “fake” palindromic reads should have their inversion site at the center of the read in cases in which they were sequenced completely (Fig. 4C). A sequencing reaction may stop at any point in a read for various reasons. If it stops after the inversion, the inversion site should be in the latter half of the sequenced read. Taken together, if a palindromic read is an artifact, the inversion site will be at the center or in the latter half of the read. We therefore investigated the relative position of the inversion site in each palindromic read for HPGP samples. As shown in Figure 4D, most of the inversion points appeared after the center, which strongly indicates that most of the palindromic reads are the result of the aforementioned artifacts. Furthermore, if palindrome reads are real, multiple reads with the same structure should be detected with sufficient sequencing depth. However, we could not find such reads with the same inversion pattern although we analyzed samples with more than 5× coverage (Supplemental Fig. S6). These results also support our inference. In addition, about two thirds of such pseudopalindromic reads showed a sudden drop in sequencing quality score around the inversion site (Fig. 4C). Some of the reads with an inversion site in the former half also showed a sudden drop in sequencing quality score, possibly meaning that they also are not real palindromes. Nevertheless, assuming that the reads with their inversion site in the first half are true palindromes, the estimated frequency of palindromic inversion is roughly one in 2000 copies. In summary, the human rDNA is a very regular array (>99.3%), and aberrant structures such as palindromes are not common.

### 45S rDNA is “all-or-none” methylated

Oxford Nanopore sequencing can also detect CpG methylation without any prior treatments. We therefore investigated the rate of CpG methylation in the human rDNA. The methylation status of each read was calculated by binning (bin size 200 nt). We used two methods for calculating the methylation frequency of 45S rDNA in each bin: first, we calculated the expected value of the proportion of methylated bases by simply taking the mean of the posterior probability of each CpG being methylated that was output by Oxford Nanopore Guppy basecaller (Supplemental Fig. S7, “average”); and second, we estimated the proportion of bases likely to be methylated by setting a threshold on the posterior probability (Supplemental Fig. S7, “threshold”). We obtained similar results by both methods; that is, the distinction between the methylated (>0.1) and less methylated (∼0.0) copies was clear in most samples except A0031 and HG03098.

**Figure 5.**
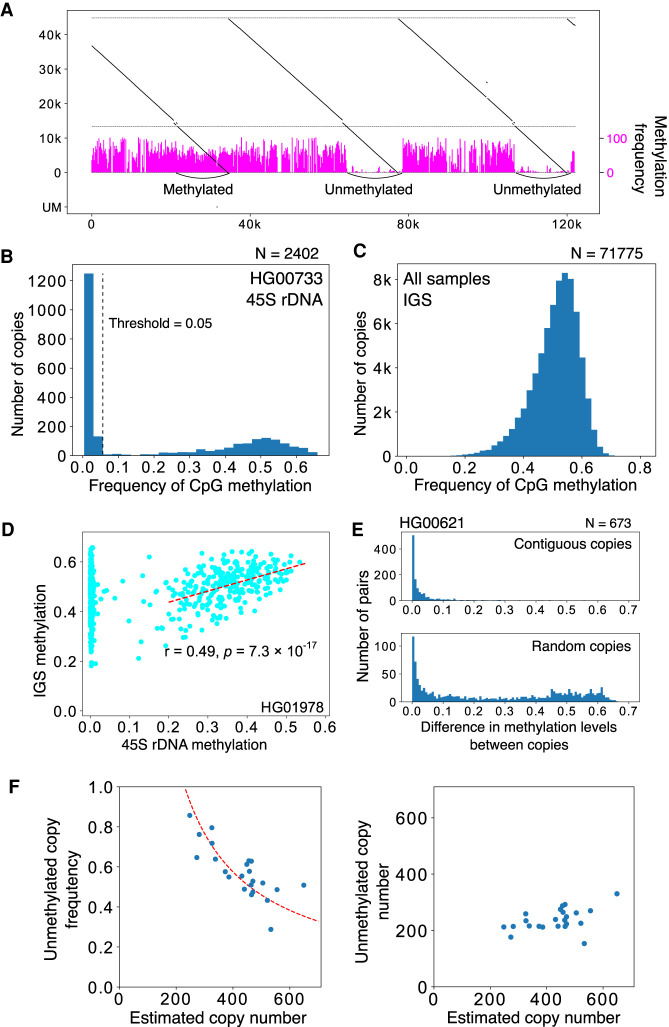
Methylation analysis of rDNA. (*A*) Representative visualization of CpG methylation. Reads are split into 200-nt bins, and the expected frequency of CpG methylation is calculated for each bin using posterior probability output by Guppy basecaller. The methylation frequency of each bin is shown as a vertical magenta bar. In the read shown, both the methylated and less methylated 45S rDNA are included. The three IGS methylation patterns are similar despite the difference in the 45S rDNAs. (*B*) Average proportion of methylated CpG for each 45S rDNA (calculated by taking the mean of posterior probabilities), and distribution in the HG00733 sample. The dashed line indicates the border between the less methylated copies and methylated copies. (*C*) Proportion of CpG methylation in the IGS in all samples. Most of the IGS copies are heavily methylated. (*D*) Methylation level of 45S rDNA and its flanking IGS. In many samples, there is a clear correlation for copies with highly methylated 45S rDNA (dashed line). (*E*) Differences in the methylation levels of contiguous 45S rDNAs and randomized controls. The methylation pattern is similar between adjacent copies of contiguous 45S rDNA, as in the case of repeat number variation in the IGS (Fig. 3C). (*F*) Relationship between the estimated rDNA copy number and the proportion of unmethylated copies (*left*) or the estimated number of unmethylated copies (*right*). The dashed line in the *left* panel is a theoretical line based on the assumption that the number of active rDNA copy is constant at 230.

We present an example of the visualization of rDNA methylation level using this method in Figure 5A. In this read, the right two 45S rDNA copies were less methylated and the left one was highly methylated. We summarized the methylation status of 45S rDNA in another sample (HG00733) (Fig. 5B). Clearly, there were methylated and less methylated copies of 45S rDNA. The less methylated 45S rDNA copies were almost methylation-free (“unmethylated”); therefore, we may say that 45S rDNAs can be classified in two states, summarized as “all-or-none methylated” (Fig. 5B). Furthermore, the unmethylated copies are thought to be transcribed (Kass et al. 1997).

### Methylation in contiguous 45S rDNA and the IGS is correlated

In contrast to coding 45S rDNA copies, the noncoding IGS seems always to be methylated based on our visualization (Fig. 5A). Therefore, we investigated methylation status in the IGSs quantitatively. Because the Butterfly/Long repeat contains microsatellites with few CpG pairs and its length is variable, we excluded this region from the analysis. As a result, we found that nearly all the IGSs are heavily methylated (average 51%) (Fig. 5C; Supplemental Fig. S8). Furthermore, we evaluated the methylation rate of 45S rDNA and its contiguous IGS and found that the rate of methylated bases in the IGS was correlated with the methylation level of contiguous 45S rDNAs in many individuals when the calculation was limited to strongly methylated 45S rDNA (>0.3), although the strength of correlation differed among samples (Fig. 5D; Supplemental Table S2). Overall, these observations suggest that heavily methylated 45S rDNA and the contiguous IGS together form heterochromatin.

### Contiguous 45S rDNAs have a similar CpG methylation pattern

Using information in large reads containing several rDNA copies, we analyzed the relationship of methylation status among contiguous 45S rDNAs (Fig. 5E; Supplemental Fig. S9). We found that contiguous 45S rDNA copies have a similar CpG methylation pattern compared with noncontiguous random copies. In other words, unmethylated 45S rDNAs form clusters. This suggests that heterochromatin structure is present in the large rDNA region and the transcription of rDNA is inhibited in this region.

We also examined how frequently adjacent copies have different methylation status in each chromosome. A previous study suggested that the transcriptional state of rDNA is determined at the chromosome level (Roussel et al. 1996). We found that a contiguous pair with different methylation status occurs at a frequency of about 1 in 20 pairs in many individuals (Supplemental Table S3). In other words, the average cluster size of methylation status is 20 copies. Therefore, for example, chromosomes that have more than 20 copies of rDNA should have, on average, more than one change in methylation status. This speculation is supported by a recent study (van Sluis et al. 2020).

### The number of R repeats is not related to rDNA methylation

Next, we tested the correlation between 45S rDNA methylation rate and the copy number of the R repeat that is associated with TTF1 (Grummt et al. 1986). Spearman's correlation between the 45S rDNA methylation rate and R repeat copy number differed among individuals (21/32 individuals showed <0.05 false discovery rate). Even in samples with a clear correlation, the tendency was not consistent among individuals, and both positive and negative correlations were observed (Supplemental Fig. S10; Supplemental Table S4). We speculate that the correlations observed in some samples were the effect of the correlation of contiguous copies; thus, they do not reflect a true correlation. Collectively, these observations suggest that the R repeat plays little role in the transcription of rRNA.

### There is no strong correlation between age, instability, and methylation

Next, we sequenced two samples from young individuals (20s) and two from older individuals (70s) to investigate age-related changes in rDNA structure (Supplemental Table S1, rows 4–7). Between the Cas9-enriched young and old samples, no large-scale structural differences were observed. This is consistent with the previous finding that aging does not increase such differences (Caburet et al. 2005).

It has been proposed that the rate of methylation of 45S rDNAs is increased with age (Wang and Lemos 2019; Watada et al. 2020). We therefore tested this relationship using the two young samples and two older samples obtained by Oxford Nanopore sequencing. However, neither the rate of unmethylated copies nor the average methylation level of methylated copies was increased in the older samples (Supplemental Table S1). We did not analyze methylation status in the same individual over time, and the number of samples was not sufficient to draw conclusions, which may explain why our results were different from previous studies.

### The higher rDNA copy number, the more methylated coding regions

We analyzed the relationship between rDNA copy number and methylation rate. To estimate the rDNA copy number per cell, we used the ratio of rDNA reads to the total reads in each sample. By this calculation, the rDNA copy number ranged from 250 to 700 copies per cell (Fig. 5F; Supplemental Table S5). These values showed good agreement with previously reported data derived by different methods, such as quantitative PCR and short-read high-throughput sequencing (Malinovskaya et al. 2018; Parks et al. 2018).

To analyze the relationship between rDNA copy number and methylation rate, we used only the HPGP data that were generated by the Human Pangenomics Reference Consortium (HPRC), which were all thought to be obtained around the same period and basecalled with Guppy 4.0.11 (Methods). This was performed to avoid artifacts caused by different library preparations and sequencing conditions. From the analysis of 23 HPGP samples, we found that the number of rDNA copies and the ratio of unmethylated copies per cell were negatively correlated (Pearson's correlation, *r* = 0.749, *P* = 1.15 × 10^−5^). In other words, the number of unmethylated copies was roughly constant irrespective of rDNA copy number per cell (Pearson's correlation, *r* = 0.07, *P* = 0.625) (Fig. 5F, right).

### rDNA instability is increased in progeroid syndrome

We also analyzed two cell lines derived from patients with progeroid syndrome, namely, Bloom syndrome patient B cells derived by EBV transformation, and Werner syndrome patient primary fibroblast cells. These syndromes are caused by mutations in the DNA repair machinery, which increases genome instability (Killen et al. 2009). A previous study using the fiber-FISH method suggested that the structure of rDNA is highly aberrant (∼50% of total rDNA copies) in Werner syndrome patients (Caburet et al. 2005). Based on the Cas9-enriched Oxford Nanopore sequencing method, the rate of noncanonical (e.g., real palindrome) copies was 1.2% and 2.4% in Werner and Bloom patient cells, respectively. These values are much higher than those in the normal samples (∼0.2%), but much lower than the previously reported value (∼50%). In both progeroid syndrome samples, we found characteristic reads, including a duplication within the 45S rDNA that may create a noncanonical rRNA structure (Supplemental Fig. S11; Supplemental Table S1). These mutations were concentrated around the 7000- to 14,000-nt region in the reference. Because they were relatively rare in the other samples (Supplemental Table S1, column 7), we speculate that they are genomic instability “hotspots,” where mutation is frequent in DNA repair compromised cells.

### Human pluripotent stem cells have a different methylation status

**Figure 6.**
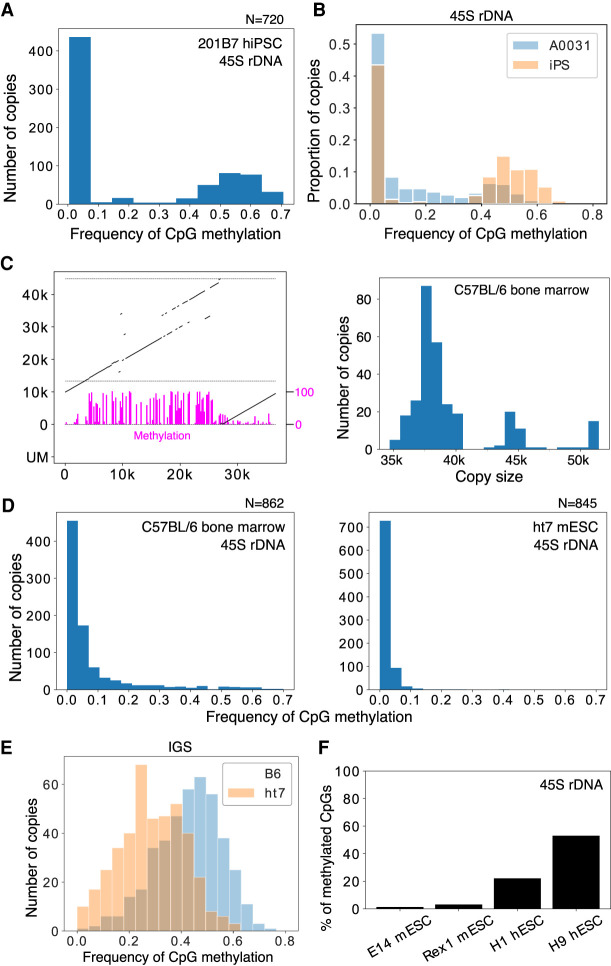
rDNA methylation in pluripotent stem cells. (*A*) The 45S rDNA methylation status of rDNA in hiPSCs does not differ from that in other differentiated samples: ∼40% of the transcribed region is methylated. (*B*) Comparison of the 45S rDNA methylation status in Werner syndrome patient fibroblasts (A0031) and iPSCs derived from them. The *y*-axis is the proportion of reads in each bin. The frequency of methylated copies is increased in iPSCs. (*C*) Representative rDNA from a Cas9-enriched Nanopore read of a mouse sample (*left*). Magenta bars represent CpG methylation. The estimated size distribution of mouse rDNA copies in C57BL/6 bone marrow cells is shown on the *right*. The rDNA copy size of mouse is much smaller than previously reported. (*D*) 45S rDNA methylation levels in B6 bone marrow cells and ht7 mESCs. Methylation levels among copies are more continuous in mice, and almost no methylation is seen in mESCs. (*E*) Comparison of IGS methylation levels in B6 bone marrow cells and ht7 mESCs. ht7 clearly shows a lower methylation level, even in the IGS region. (*F*) Proportion of CpGs methylated in the 45S rDNA of mESCs and hESCs determined by using publicly available short-read bisulfite whole-genome sequencing data. Although both samples of mESCs show a very low level of methylation, a substantial proportion of CpGs are methylated in hESCs.

Next, we analyzed rDNA methylation status in human induced pluripotent stem cells (hiPSCs, 201B7) by Cas9-enriched sequencing. In ESCs and iPSCs, rDNA is thought to be globally unmethylated because of the high transcription activity (Gupta and Santoro 2020). However, around half of the 45S rDNAs were methylated and the IGS was heavily methylated in the iPSCs, similar to differentiated cells (Fig. 6A; Supplemental Fig. S12; Discussion). We also tested iPSCs derived from Werner syndrome patient fibroblast cells (A0031) (Shimamoto et al. 2014). Similarly in these iPSCs, a substantial proportion of 45S rDNAs were methylated and the frequency was higher than in the original A0031 fibroblast cells (52% vs. 43%, *P* = 1.2 × 10^−6^, Fisher's exact test) (Fig. 6B), in contrast to the results of previous studies (Woolnough et al. 2016; Wang and Lemos 2019).

In terms of rDNA stability, the frequency of aberrant structures was found to be significantly decreased in A0031-derived iPSCs (*P* = 0.007, Fisher's exact test) (Supplemental Table S1). This is consistent with the finding that iPS induction suppresses chromosomal instability (Shimamoto et al. 2014), and we speculate that cells with stable genetic information are selected during the iPS induction process. If this is the case, the increased methylation of the transcribed region might be a result of the selection process.

### rDNA structure and methylation in the mouse

To test the generality of the Nanopore sequencer, we also analyzed two mouse-derived samples (bone marrow cells extracted from the femur of an 8-wk-old male C57BL/6J mouse and feeder-free ht7 embryonic stem cells [ESCs] derived from 129/Ola strain) (Niwa et al. 2000). Although mouse ribosomal DNA has been said to be around 45 kb in length (Grozdanov et al. 2003), we found that the average estimated size was much shorter than previously reported at 39.5 and 38 kb for C57BL/6J and ht7, respectively. Mouse rDNA also has variation in the repeat number in the IGS, and this seems to determine the size distribution of rDNA (Fig. 6C; Supplemental Fig. S12).

In terms of methylation, 45S rDNAs of mESCs were almost completely free from methylation, which is comparatively different from hiPSCs (Fig. 6D, right). In bone marrow cells, some of the 45S rDNAs were clearly methylated but, unlike in human, the distribution of CpG methylation among copies was continuous rather than bimodal, making it difficult to clearly define methylated copies (Fig. 6D, left). The IGS was also methylated in mouse, but the frequency of methylation was much lower in mESCs than in hiPSCs (Fig. 6E). To examine whether the difference in methylation status between hiPSCs and mESCs was the result of the derivation method (i.e., ES vs. iPS) or related to species differences (i.e., human vs. mouse), we reanalyzed publicly available short-read-sequencer data, which showed that 45S rDNAs of mESCs are rarely methylated but a substantial proportion of CpGs in hESCs are methylated (Fig. 6F). Thus, the difference is likely to be ascribed to species variations. In fact, mESCs are known to be in a more undifferentiated state compared with hESCs and show global hypomethylation (Nichols and Smith 2009; Nishino and Umezawa 2016).

## Discussion

In this study, we analyzed the long rDNA array using data from the Oxford Nanopore sequencer. Our findings provide a new picture of the rDNA structure based on 39 samples with many reads that were directly extracted from human cells (∼78,000 copies, more than 3 billion bases).

By using a dot plot method that visualizes multiple copies of rDNA, we first characterized the rate of large-scale rDNA instability (inversion, deletion, and other noncanonical structures) and found that such mutations are relatively rare, contrary to a previous report based on the fiber-FISH method (Caburet et al. 2005). We also showed that Oxford Nanopore sequencing occasionally produces artifactual palindromic reads that are considered to be difficult to distinguish from true palindromic reads (de Lannoy et al. 2017). We found such artificial palindromic reads have characteristic features, such as a poor quality score around the inversion and the position of the inversion site, and succeeded in recognizing them. As a result, we conclude that real palindromic structures are relatively rare. Last, we found that, although rDNA instability was not increased by age in our samples, it was increased in cells from patients with progeroid syndrome. In our study, approximately 40 samples were analyzed, and only 0.2% (on average) of structures were noncanonical. This value is much lower (about 1/150) than the value previously reported using fiber-FISH (Caburet et al. 2005). Thus, we conclude that the human rDNA is a relatively regular array. We do not know what caused the difference in the rate of noncanonical copies by these two methods. As the rate of noncanonical copies in Werner patient cells was higher using both methods, the fiber-FISH method may have a higher background.

We found that the R and Butterfly/Long repeat regions are variable in different copies, although they are similar in contiguous copies. Because the R and Butterfly/Long repeat regions have small repetitive sequences within the repeats, they may form a secondary DNA structure and inhibit the replication fork to induce instability. The R repeat contains many copies of the Sal box associated with TTF1 (Fig. 1A), which is known to arrest the replication fork both in vivo and in vitro (Santoro and Grummt 2005; Akamatsu and Kobayashi 2015). This sequence may work as a recombination hotspot, as observed in budding yeast (Supplemental Fig. S1; see below).

In contrast to variation, our study indicated that there is structural similarity between IGSs in contiguous copies. This suggests that gene conversion takes place frequently in the human rDNA, as in the budding yeast rDNA (Ganley and Kobayashi 2011). Previous studies also have suggested the possibility of IGS homogenization within the same chromosome (Gonzalez and Sylvester 2001). Our study strongly supports this view.

We also found that IGSs are classified into two types among individuals (Fig. 3B; Supplemental Fig. S3). The mechanism behind this bimodal distribution of Butterfly/Long repeat copy number is a fascinating issue to be addressed in future studies. Another mystery is the presence of a rare type of IGS, which was observed in many samples. If the efficiency of gene conversion is high, such copies should be excluded. One explanation for the rare-type IGS is that these variations are often generated by chance and resolved over time by homogenization. We are not sure when such homogenization occurs in the rDNA. In other words, we cannot tell whether the main source of the variations described in our study were germline or somatic mutations. To address the question, we would need to compare the variations in different tissues of the same individual. However, in terms of Bloom and Werner syndrome cells, their rDNA instabilities are most likely caused by somatic mutations when the repair efficiency is reduced by the combination of recessive alleles.

In the budding yeast, it is known that gene conversion occurs frequently, and all copies essentially have the same sequence in a cell (Ganley and Kobayashi 2007). As a mechanism, Fob1/RFB-dependent rDNA recombination seems to be important (Ganley and Kobayashi 2011). As mentioned above, such an RFB site is also present in the R repeat in human rDNA. Therefore, a similar recombination repair system may also contribute to sequence homogenization in human cells (Akamatsu and Kobayashi 2015). Moreover, in progeroid syndrome patient cells, in which the activity of DNA repair is reduced, the number of noncanonical copies increased. Such rDNA instability has been also observed in the yeast WRN homolog mutant *sgs1* (Sinclair and Guarente 1997).

In terms of rDNA methylation, we found that there is an obvious difference between methylated and unmethylated 45S rDNAs (Fig. 5A,B). In unmethylated 45S rDNAs, the methylation rate was close to 0 such that the genes are likely to be transcribed (for review, see Kass et al. 1997). The methylation status was also similar in contiguous copies. This may be because heterochromatin forms around these regions and affects rDNA silencing. In contrast to the “all-or-none” methylation pattern of 45S rDNAs, the noncoding IGS regions were always methylated, and the level was correlated with the methylation level of the contiguous 45S rDNAs (Fig. 5D). These observations suggest that the IGS is always in a similar heterochromatin structure and that 45S rDNA activation affects the region. We also must note that, because all the IGS regions in the rDNA are heavily methylated, the transcriptional rate of noncoding sequences in these regions may not be very high. Therefore, if this is the case, at least some noncoding transcripts are likely to come from rDNA fragments that are scattered all over the human genome (Cherlin et al. 2020).

We found that unmethylated copies are negatively correlated with total rDNA copy number in a cell, suggesting that the number of unmethylated copies is roughly constant in different individuals, at least in the same tissue. This finding also supports our definition of unmethylated copies and our view that they are the actively transcribed ones. One possible mechanism behind the regulation of active copy number is that the transcription factor dosage is limited. Alternatively, the volume of the nucleolus fibrillar center may be kept at a constant level, which restricts the number of rDNA copies that can be held inside. Further analysis will be required to reveal the underlying mechanism.

In terms of mouse rDNA, we found that some features were similar to the human rDNA, such as repeat number variation in the IGS. One clear finding was that the unit length was shorter (∼39 kb) than reported previously (∼45 kb) (Grozdanov et al. 2003). The reason might be the difficulty in assembling sequences filled with repeats using relatively short reads.

Last, regarding the methylation in ES and iPS cells, the results differed between human and mouse. We expected that both cell types would be less methylated than their differentiated counterparts because the nucleolus of ES and iPS cells is known to be larger and rRNA transcription activity is high (Woolnough et al. 2016; Wang and Lemos 2019; Gupta and Santoro 2020). In the human iPSC and ESCs, however, the methylation status of the 45S rDNA was similar to that in differentiated cells. In contrast, most of the 45S rDNA copies in the mouse ESCs were unmethylated. This difference between mouse and human is thought to stem from the difference between their developmental stages. Mouse ESCs are always in a pre-X-Chromosome-inactivated status and are globally hypomethylated; in human ESCs, in contrast, the X Chromosome is already inactivated in many cases, and the other genomic regions are also highly methylated (Nichols and Smith 2009; Tomoda et al. 2012; Nishino and Umezawa 2016).

To date, we do not know whether each chromosome has common transcription status features. In the future, if the average sequencing length of Nanopore sequencing becomes longer and the sequencing quality becomes higher, it would be possible to connect the reads and to reconstruct chromosome level rDNA structure.

In summary, our results have revealed several new aspects of the most highly transcribed housekeeping gene, rDNA, in terms of its stability, structure, and methylation status.

## Methods

### DNA extraction

DNA was extracted by modified Sambrook and Russell DNA extraction. In brief, 1 × 10^6^ cells were pelleted, and 500 µL of TLB was added (10 mM Tris-Cl at pH 8.0, 25 mM EDTA at pH 8.0, 0.5% [w/v] SDS, 20 µg/mL RNase A). After mixing by inversion and incubating for 1 h at 37°C, 1 µL of 20 mg/mL Proteinase K (Roche) solution was added, and the mixture was further incubated for 3 h at 55°C. Next, 500 µL of TE-saturated phenol was added, and the solution was rotated until the water phase was clear. Phase lock gel (Dow Corning high vacuum grease) was added, followed by centrifugation for 5 min, and then the upper phase was decanted into a 1.5-mL tube. Phenol/chloroform/isoamyl alcohol (25:24:1) was added, and the preceding procedure was repeated. The resultant solution was placed in a 5-mL tube, and 200 µL of 5 M ammonium acetate and 1.5 mL 100% ethanol were added with gentle rotation at RT until the solution was homogeneous. The DNA precipitate was collected by pipetting and placed in a 1.5 mL tube containing 70% ethanol. After centrifugation, 50 µL of TE was added to the pellet, which was left overnight at 4°C. DNA concentration was measured with a Qubit assay kit (Invitrogen).

### Construction of the Cas9-enriched Oxford Nanopore library

The published protocol (Gilpatrick et al. 2020) was modified specifically for rDNA, which consists of hundreds of copies. First, Cas9 RNP was assembled as described previously. Then, 500 ng of DNA was dissolved in 9 µL of 1× CutSmart buffer (New England Biolabs) and sheared by pipetting 30 times with a pipette set at 8 µL with a P10 tip. One microliter of Quick CIP (New England Biolabs) was added, and the solution was incubated for 10 min at 37°C, followed by 2 min at 80°C. Next, 0.5 µL of RNP, 0.3 µL of 10 mM dATP and 0.3 µL of Taq DNA polymerase were added to the solution before incubation for 15 min at 37°C and for 5 min at 72°C. The ligation mix (5 µL of LNB, 3 µL of Quick ligase [New England Biolabs], 1.2 µL of MQ, 0.8 µL of AMX) was then added to the DNA solution in two stages, with tapping to mix between additions. After incubating for 10 min at room temperature, 2.7 µL of 5 M NaCl was added, followed by incubation for 5 min. After centrifuging at 15,000 rpm for 5 min, the supernatant was removed, and 100 µL of 4.5% PEG 6000, 0.5 M NaCl, 5 mM Tris-HCl (pH 8.0) were added. After centrifuging again for 1 min, the supernatant was removed and the pellet was dissolved in 10 µL of EB. In our experience, centrifugation with salt rather than Ampure beads resulted in a higher library yield, and a shorter centrifugation time was preferable. It was extremely rare to find reads containing more than two copies of rDNA with this method, indicating that the in vitro Cas9 efficiency is sufficient. The four gRNA target sequences were 5′-ATGAACCGAACGCCGGGTTAAGG, 5′-AGGACGGTGGCCATGGAAGTCGG, 5′-ACCTCCACCAGAGTTTCCTCTGG, and 5′-TATCCTGAGGGAAACTTCGGAGG.

### Mice

Eight-week-old C57BL6/JJc1 mice were purchased from CLEA Japan, Inc. (Tokyo, Japan). Bone marrow cells were extracted as described previously (Madaan et al. 2014). All experiments were approved by the Animal Experiment Ethics Committees of the University of Tokyo (Experiment No. 0210) and performed in accordance with the provided manual.

### Cell culture

EBV-transformed B cells were obtained from National Institutes of Biomedical Innovation, Health and Nutrition. 201B7 hiPSCs were obtained from Riken BioResource Research Center. EBV-transformed B cells were cultured as a floating culture in T25 flasks containing RPMI-1640 supplemented with 10% FBS. hiPSCs were cultured on vitronectin-coated plates with AK02N medium. ht7 mESCs were cultured on 0.1% gelatin-coated plates with standard GMEM-based medium (10% FCS, 1 × NEAA, 1 mM sodium pyruvate, 10^–4^ M 2-ME, 1000 units/mL mLIF). A0031 Werner syndrome patient cells and the iPSCs derived from them were cultured as described previously (Shimamoto et al. 2014).

### Screening of rDNA-derived Oxford Nanopore reads

To analyze the HPGP whole-genome sequencing samples, we downloaded the FAST5, FASTQ, and sequencing summary files. First, based on the sequencing summary file, we excluded reads that did not pass the sequencing quality filtering. Next, each read was split and mapped to the rDNA reference file (KY962518.1) by using BWA-MEM (v0.7.17) and the “ont2d” option. We only used reads that had >40,000 nt of continuous rDNA region at either end. Moreover, to remove reads derived from a microsatellite stretch similar to that included in the IGS of rDNA, we checked whether the reads contained at least 10% of split reads that mapped to the coding region.

### Visualization of the rDNA-derived Oxford Nanopore reads

Each FASTQ read was split into smaller reads of 300 nt, and mapped to the rDNA reference sequence by using BWA-MEM (v0.7.17) as described above. The split reads were then visualized as lines based on their position in the original read. To visualize the Phred quality score, the score was binned in 200-nt bins and the mean score was plotted. Visualization of CpG methylation was performed similarly by binning reads in 200-nt bins. For each bin, the frequency of methylation was calculated based on the “average” (see below), and the value was plotted as a bar.

### Finding noncanonical copies

First, the read was split and mapped to the rDNA reference sequence. If >10% of the mapped segment was in the opposite direction to the dominant direction, the read was classified as inverted and plotted. If the distance between each mapped read differed from the expected length by >500 nt, the reads were plotted as potential reads containing noncanonical copies. If both of two neighboring reads were within the R repeat or Butterfly/Long repeat region, we did not count them as aberrant copies owing to the natural variation in these regions. Each plotted read was then manually classified based on the visualization.

### Estimation of repeat length

Using BWA-MEM software, 500-nt rDNA sections located at 10,000, 20,000, and 30,000 nt in the reference sequence were mapped to each read. Next, the distance between the mapped positions of 500-nt sections at 10,000 and 20,000 in each read was used to estimate the R repeat length, and the distance between the mapped positions of 20,000 and 30,000 was used to estimate the Butterfly/Long repeat length. Because genomic mutations in rDNA are rare, most of the variations obtained by this method should be caused by repeat length variation in the repeat regions.

### Methylation analysis

For the reads that were thought to contain rDNA, FAST5 files were extracted by using ont_fast5_api and basecalled by using Guppy basecaller v4.2.2 with dna_r9.4.1_450bps_modbases_dam-dcm-cpg_hac_prom configuration. For threshold-based methylation analysis, we used 0.8 as the threshold for posterior probability. In the HPGP database, there are two types of Nanopore data, which are labeled as NHGRI-UCSC and HPRC. For the analysis comparing transcribed region methylation frequency and rDNA copy number, we used only data labeled as HPRC because they were submitted to the database over a short period of time and thus were likely to be less affected by differences in experimental conditions. The number of samples available was sufficient for the analysis (23 samples).

### Whole-genome bisulfite sequencing analysis

FASTQ files were first cleaned up with Trim Galore! (v0.6.6) to remove adapters (Martin 2011). The frequency of CpG methylation in the rDNA coding region was then estimated by Bismark software (v0.22.3) (Krueger and Andrews 2011) and Bowtie 2 (v2.3.5) using the reference genome that contained only the rDNA coding region sequence (Langmead and Salzberg 2012). We used the following data: m14 mESC (SRR610046, SRA), Rex1 mESC (SRR5099302, SRA), H1 hESC (ENCFF311PSV, ENCODE Project), and H9 hESC (ENCFF384QMG, ENCODE Project). Files are available through the NCBI Sequence Read Archive (SRA; https://www.ncbi.nlm.nih.gov/sra/) and ENCODE (https://www.encodeproject.org/files/), respectively.

## Data access

All raw Cas9-enriched sequencing data generated in this study have been submitted to the NCBI BioProject database (https://www.ncbi.nlm.nih.gov/bioproject/) under accession numbers PRJNA745386 and PRJNA745391.

## Supplementary Material

Supplemental Material
