# Supplementary material for: The human ribosomal DNA array is composed of highly homogenized tandem clusters
Source: Genome Res. 2021 Nov;31(11):1971–82. doi: 10.1101/gr.275838.121 (PMC8559705; doi:10.1101/gr.275838.121)
Supplement: Supplemental Material [file supp_31_11_1971__DC1.html]

The human ribosomal DNA array is composed of highly homogenized tandem clusters — Supplemental Material 

# The human ribosomal DNA array is composed of highly homogenized tandem clusters

## Supplemental Material

- Supplemental\_Fig\_S1-S13.pdf
- Supplemental\_Table\_S1.xlsx
- Supplemental\_Table\_S2.xlsx
- Supplemental\_Table\_S3.xlsx
- Supplemental\_Table\_S4.xlsx
- Supplemental\_Table\_S5.xlsx
