# Supplementary material for: The human ribosomal DNA array is composed of highly homogenized tandem clusters
Source: Genome Res. 2021 Nov;31(11):1971–82. doi: 10.1101/gr.275838.121 (PMC8559705; doi:10.1101/gr.275838.121)
Supplement: Supplemental Material [file supp_gr.275838.121_Supplemental_Fig_S1-S13.pdf]

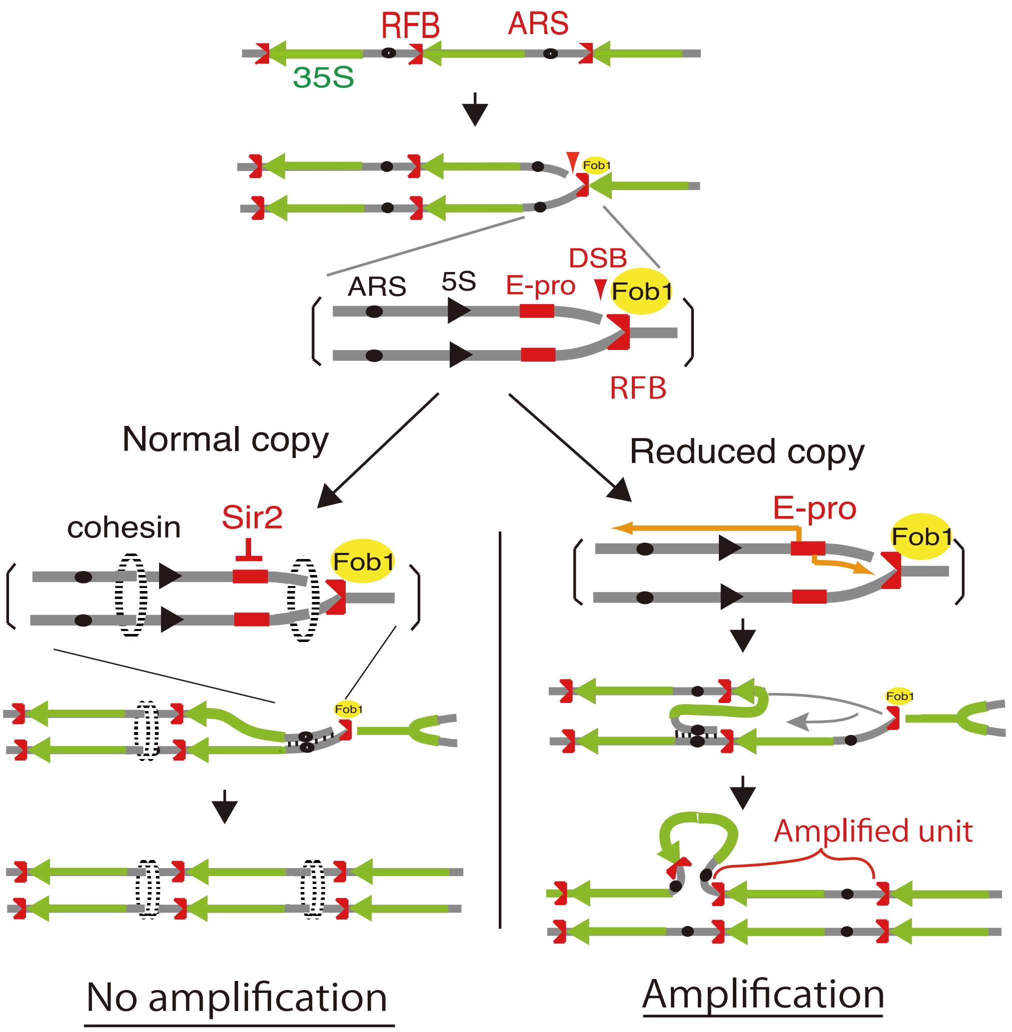

Supplemental Figure S1.

rDNA recombination for amplification in budding yeast.

Three copies of rDNA are shown. Green arrows represent the 35S rDNA and black arrowheads represent the 5S rDNA. The replication fork barrier (RFB), replication origin (ARS) and non-coding promoter (E-pro) are shown. Fob1 inhibits the replication fork and induces a DNA double strand break to trigger amplification of rDNA. Sir2 represses E-pro and ensures cohesin association, which inhibits unequal sister chromatid recombination for amplification.

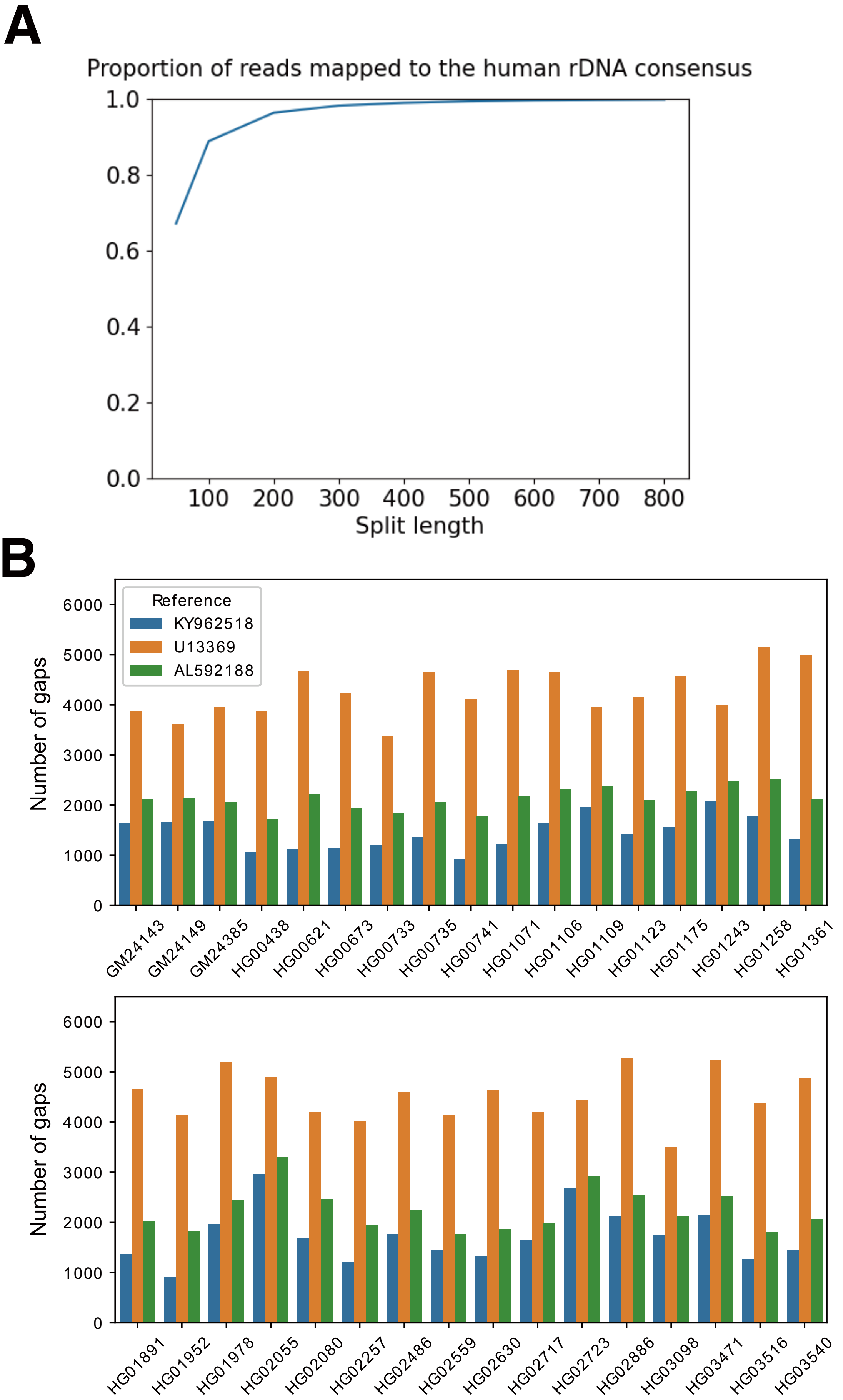

Supplemental Figure S2

(A) Proportion of Nanopore reads that mapped to the human rDNA consensus sequence by split length. Longer split length resulted in higher mapping frequency.

(B) Split reads mapped to three different reference rDNA sequences. The distances between the mapped positions of successive split reads were calculated. If the distances differed from the expected distance by more than 100 nt, they were counted as "gaps". We counted the number of these gaps to determine which reference was the most similar to actual rDNA sequences.

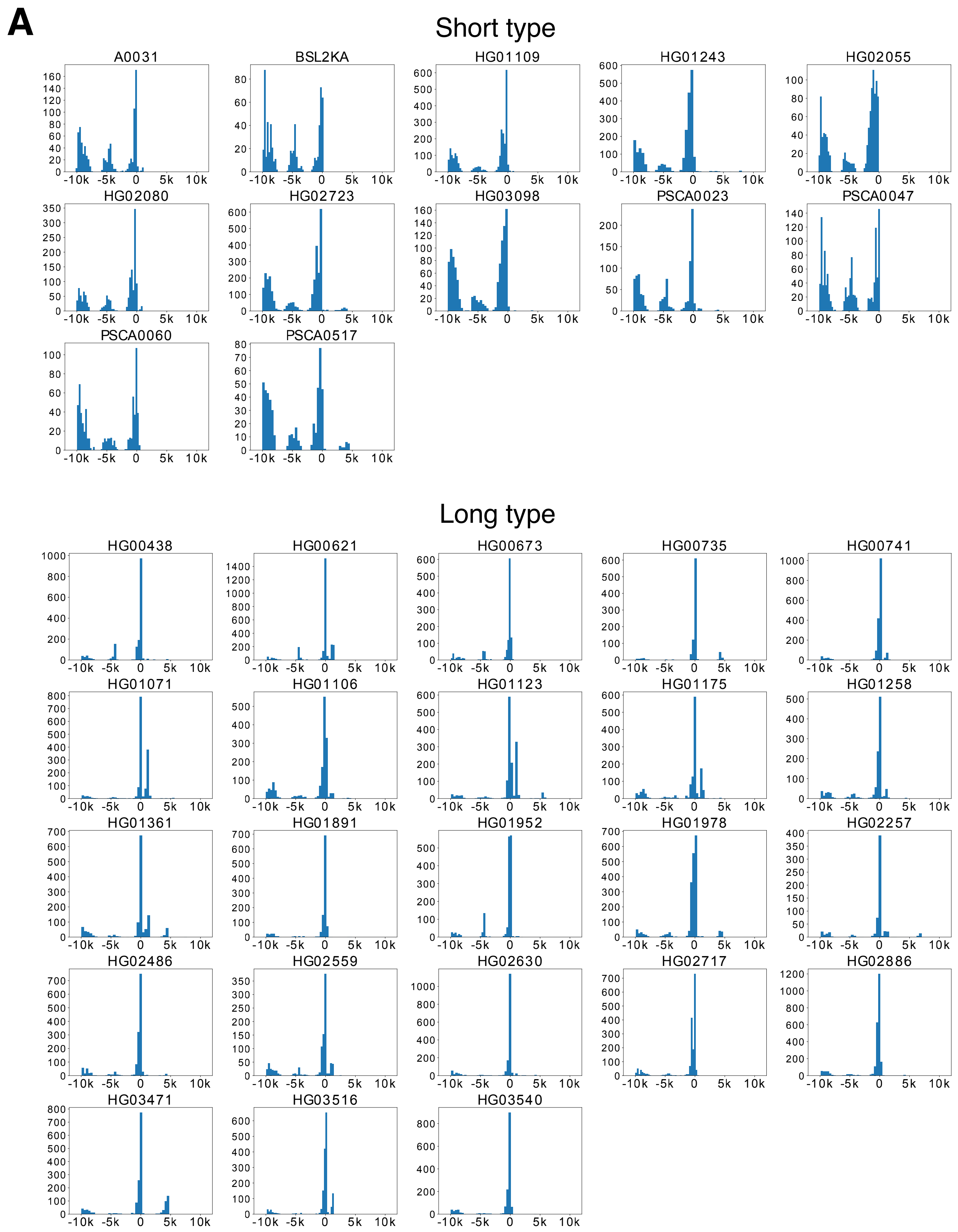

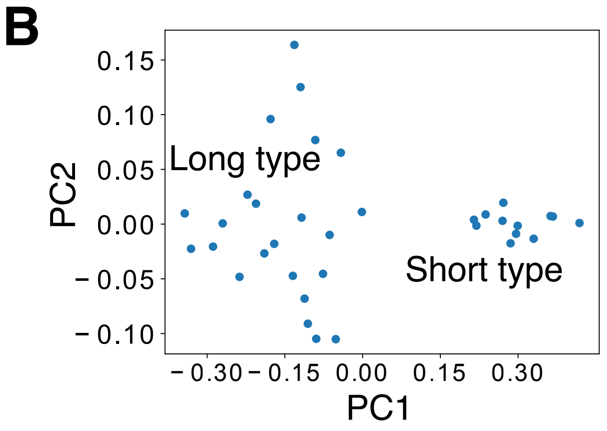

Supplemental Figure S3

(A) Length distributions of the Butterfly/Long repeat region in various samples. The distribution could be roughly classified into two types (short and long) based on average length with each type sharing a similar distribution.

(B) Principal component analysis (PCA) of the Butterfly/Long repeat distribution. Each distribution was vectorized by binning by repeat length and by calculating the proportion of copies falling into each bin. The distributions could clearly be distinguished simply by the first component.

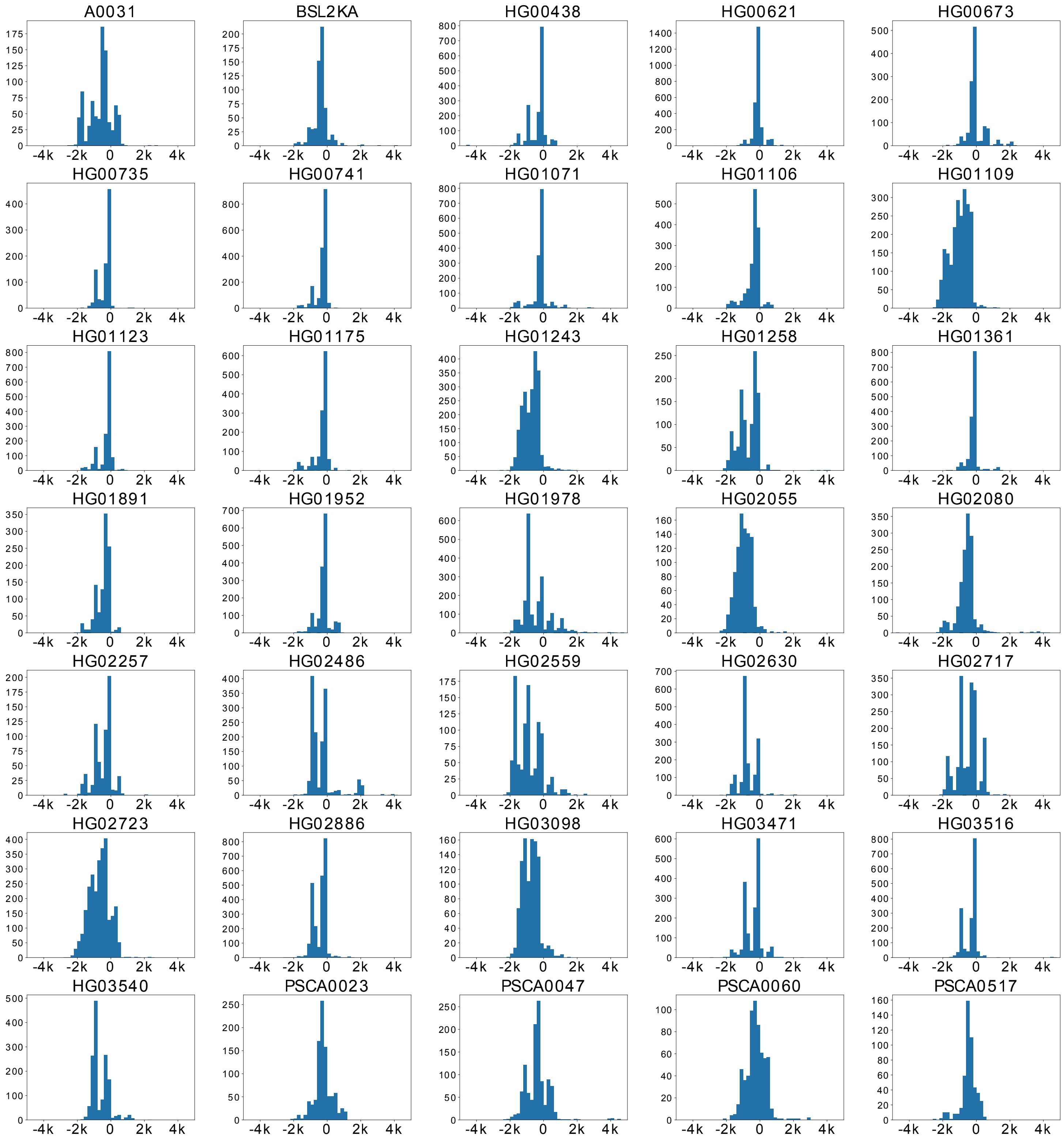

Supplemental Figure S4

Length distribution of the R repeat region in various samples. Peaks corresponding to different numbers of repeats are evident in some samples, while the distribution is more continuous in others. Overall, there is a considerable variation among samples.

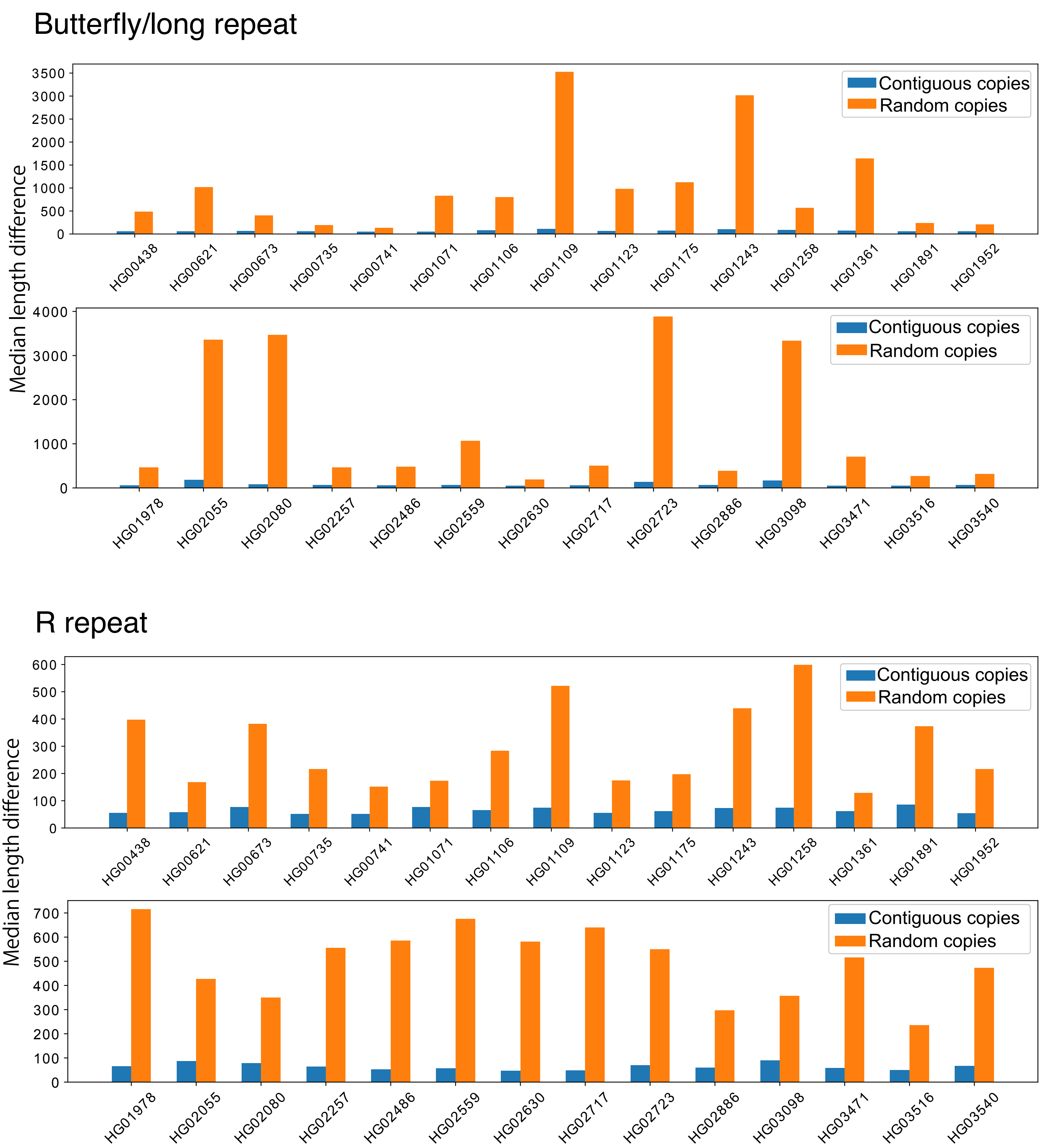

Supplemental Figure S5

Comparison of the median difference in length of the Butterfly/long repeat and R repeat between contiguous and random copies. Contiguous copies are clearly similar to each other.

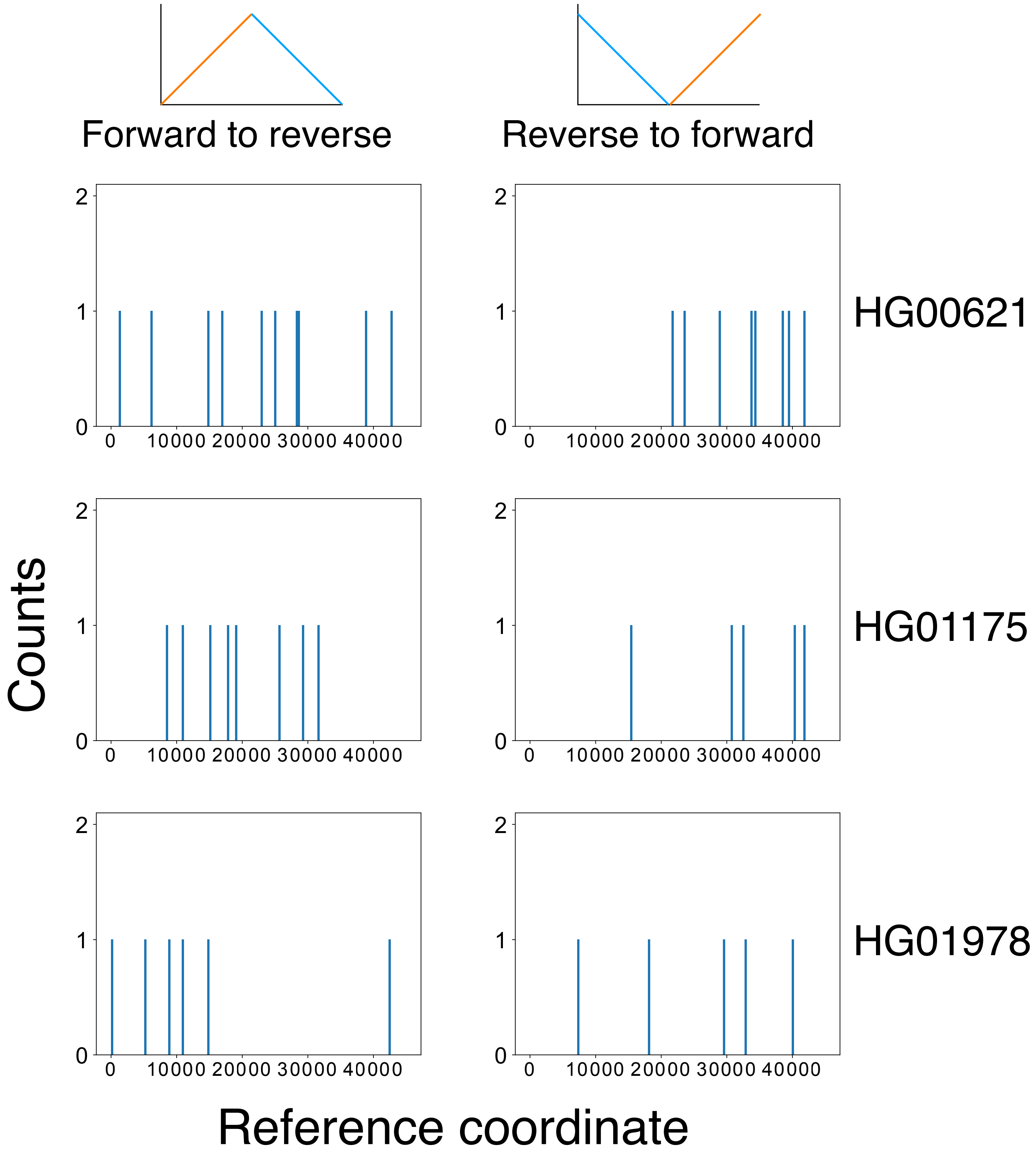

Supplemental Figure S6

Palindromic inversion points along the rDNA reference for samples with >5x coverage. As palindromes can take two directions, we divided the distributions into "forward to reverse" and "reverse to forward". In all samples, the same inversion locations were rarely seen, indicating that they are artifacts.

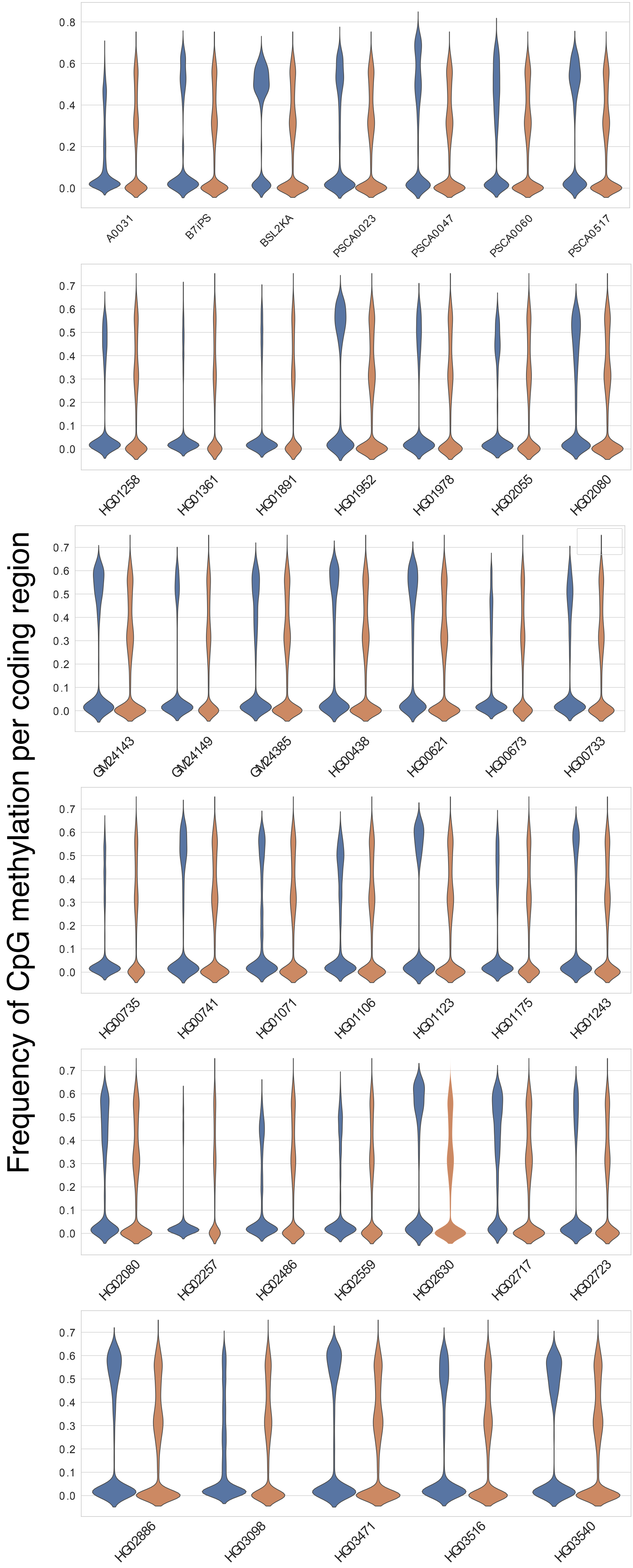

Supplemental Figure S7
Calculation of the frequency of CpG methylation in the rDNA coding region by two methods. In the “average” method (blue), we simply took the mean of the posterior probability; in the “threshold” method (orange), we set the threshold of the posterior probability at 0.8 and calculated the proportion of methylated CpGs. In most cases, methylated (>0.2) and less methylated (~0.0) copies could easily be distinguished no matter which method was used. Note that here we used a violin plot, which shows data summarized by kernel density estimation; therefore, the distribution looks more continuous than the actual histograms shown in the main manuscript (e.g., Fig. 4B).

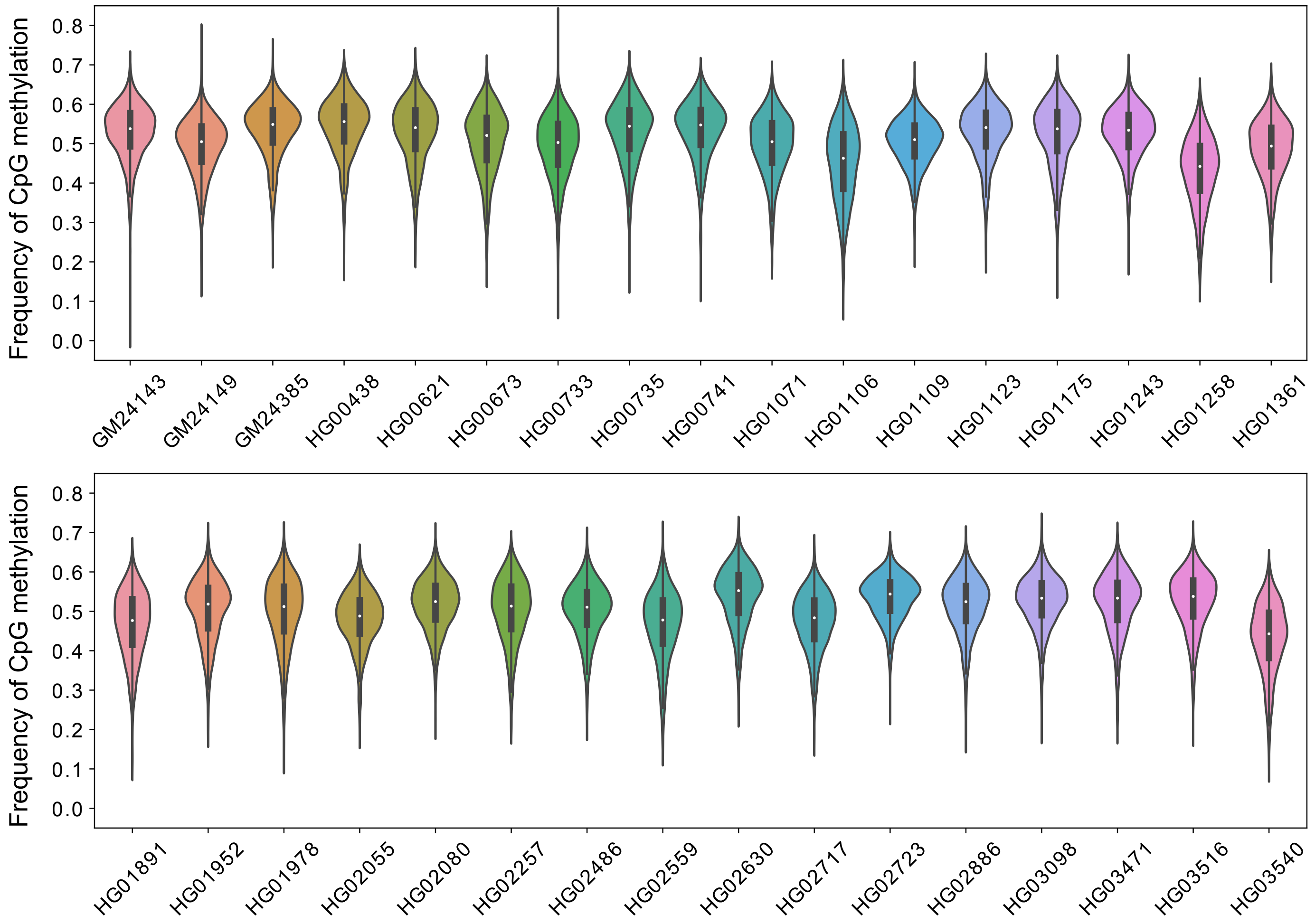

Supplemental Figure S8

Methylation levels of the IGS region in different individuals. The frequency of methylation in all copies was above that of the 45S unmethylated cutoff, 0.05.

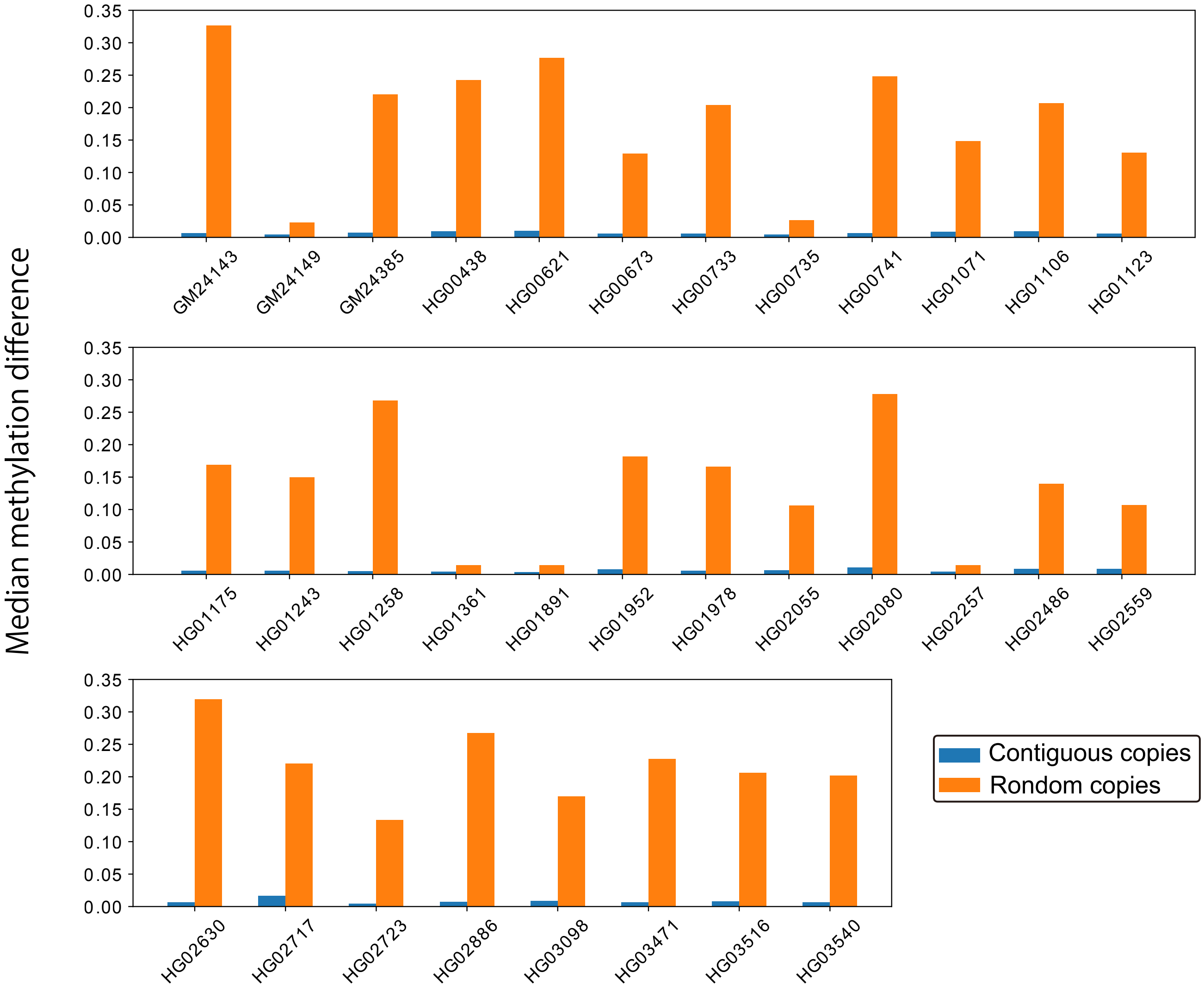

Supplemental Figure S9

Comparison of the median difference in CpG methylation level in 45S rDNA between contiguous and random copies. Contiguous copies share a similar methylation level regardless of sample.

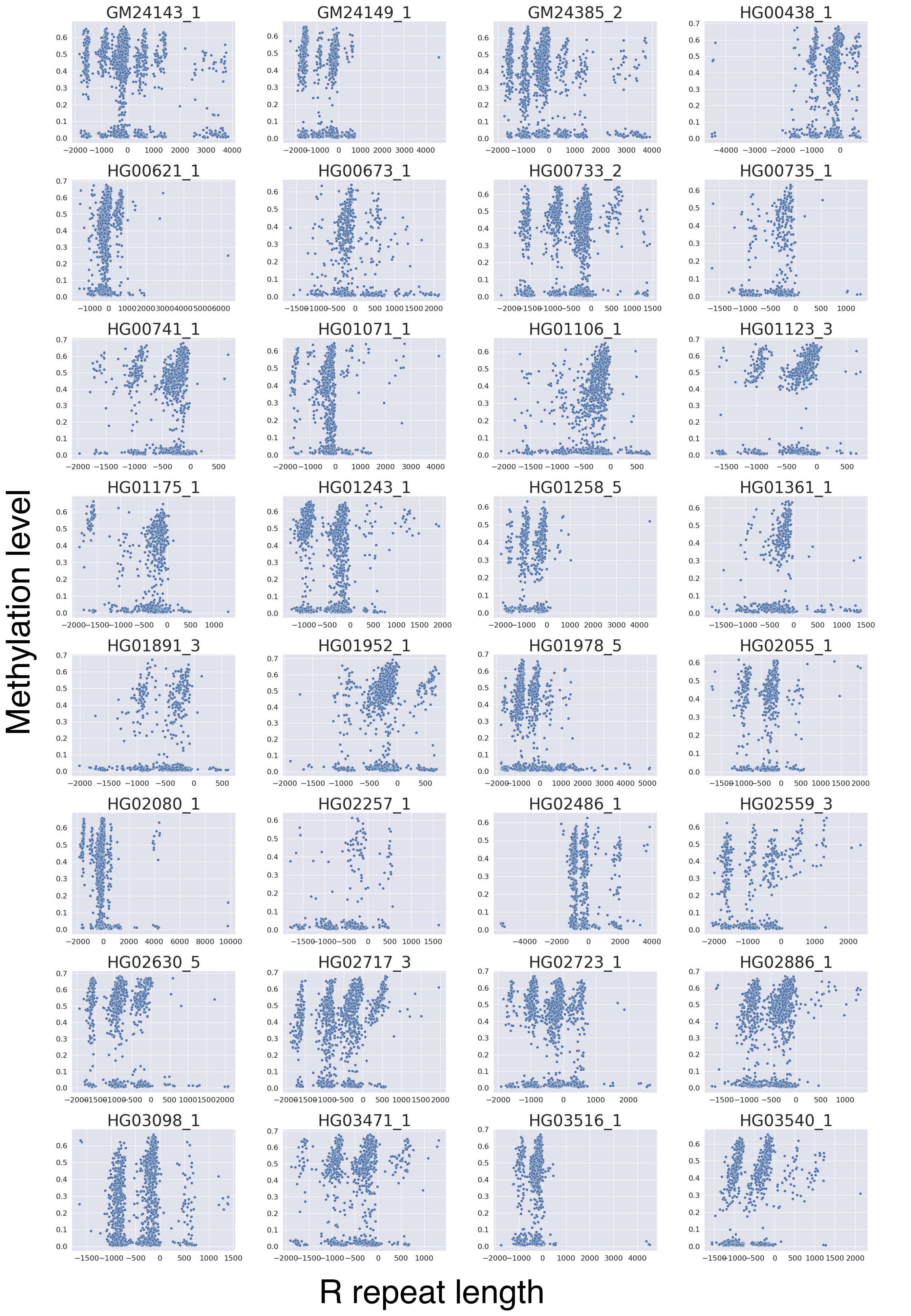

Supplemental Figure S10

Relationship between the methylation level in 45S rDNA and the length of contiguous R repeats.

There may be some correlations, but they differ among individuals; that is, there is no consensus relationship.

Therefore, any apparent correlations may be byproducts of correlations between contiguous copies.

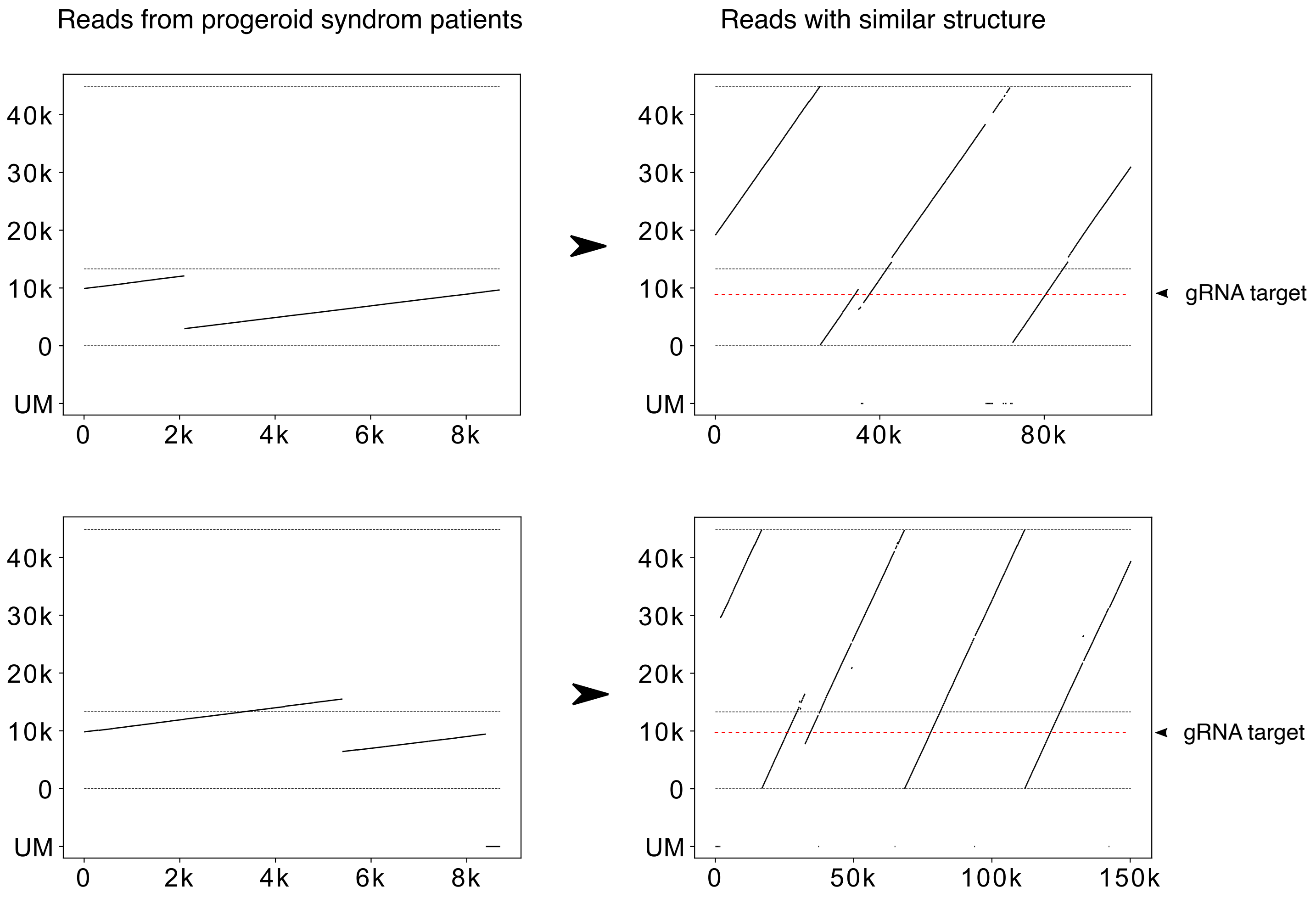

Supplemental Figure S11

(Left panels) Reads from Cas9-enriched samples. (Right panels) Examples of WGS reads with mutations that have recombinational hotspots similar to those seen in Cas9-enriched progeroid syndrome samples. UM: unmapped

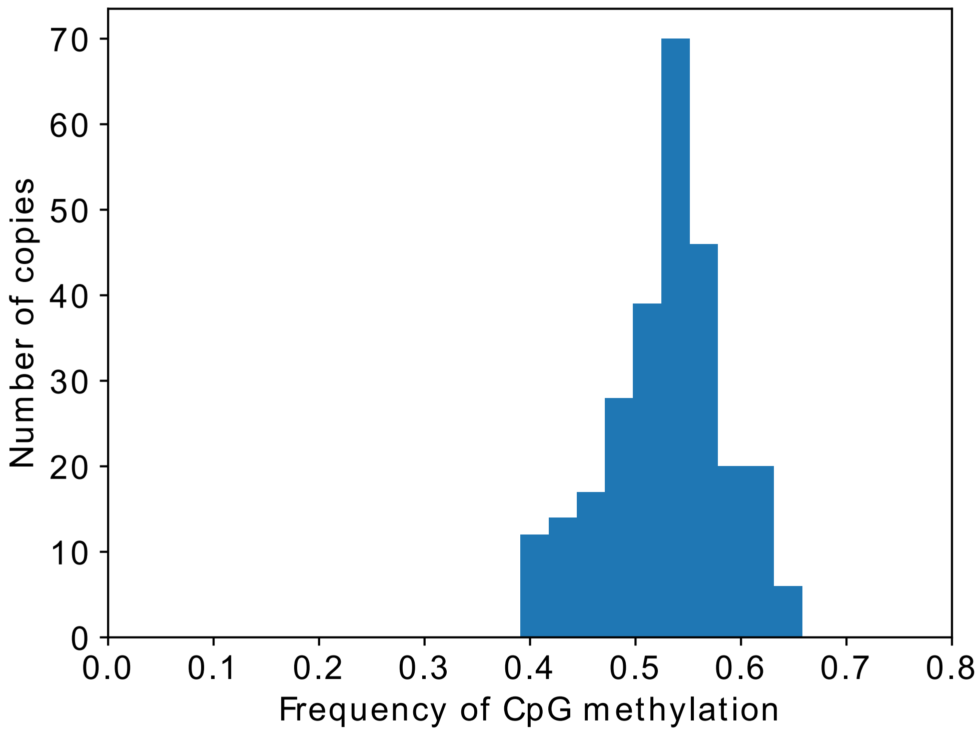

Supplemental Figure S12

Methylation level of the IGS in 201B7 hiPSCs.

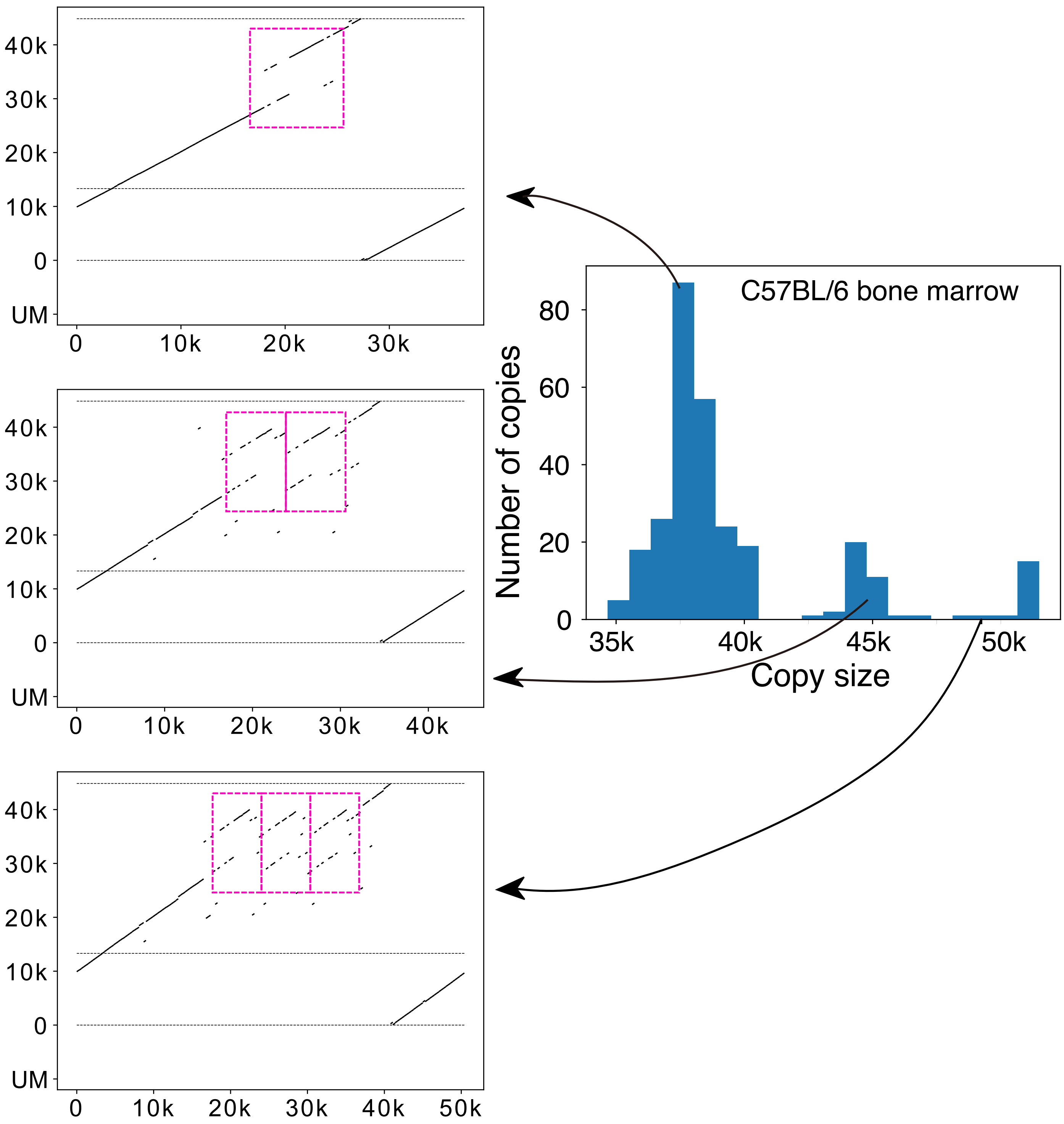

Supplemental Figure S13

Distribution of rDNA copy size in mouse C57BL/6J bone marrow cells and three representative visualizations of the rDNA in each peak. Repeat units in the IGS are indicated by dashed squares.
